# Economic analyses of freestyle libre systems for people living with diabetes: a systematic literature review

**DOI:** 10.1186/s12962-025-00673-1

**Published:** 2025-11-19

**Authors:** Gerard de Pouvourville, Jack Timmons, Fleur Levrat-Guillen, Anthony Zara, Yeesha Poon

**Affiliations:** 1 Department of Economics, ESSEC Business School, Cergy-Pontoise, France; 2 Formerly of Abbott Diabetes Care, Alameda, CA USA; 3 Abbott Diabetes Care, Maidenhead, Berkshire, UK; 4 Formerly of EVERSANA, Burlington, ON Canada; 5 Abbott Diabetes Care, 1420 Harbor Bay Parkway, Alameda, CA 94502 USA

**Keywords:** Continuous glucose monitoring, Cost effectiveness, Economic evaluation, FreeStyle Libre, Systematic literature review, Type 1 diabetes mellitus, Type 2 diabetes mellitus

## Abstract

**Introduction:**

This study systematically reviewed economic evaluations of FreeStyle Libre systems (FSL) for glucose monitoring among people living with type 1 (T1DM) or type 2 diabetes mellitus (T2DM).

**Methods:**

Systematic searches were conducted on 27 February 2024 using the MEDLINE, Embase, and Cochrane databases. Study selection was conducted by two reviewers who independently reviewed titles, abstracts, and full article texts. Study quality was assessed using the Consolidated Health Economic Evaluation Reporting Standards 2022 checklist.

**Results:**

In total, 18 cost-effectiveness studies were included; 17 were based on data for the original FSL device, with only one using FSL2 data. There were no major issues with study quality. The ten studies comparing FSL with self-monitoring of blood glucose (SMBG) reported FSL to be cost effective in populations with T1DM (seven studies) or T2DM (six studies). Eight studies reported other sensor-based systems to be cost effective versus FSL.

**Conclusions:**

Existing evidence suggests FSL systems are cost effective versus SMBG among people living with T1DM or T2DM on intensive insulin. Additional studies are needed to compare FSL systems with other sensor-based systems, considering the range of available evidence, the appropriateness of the compared devices, and the selection of modelling assumptions.

**Supplementary Information:**

The online version contains supplementary material available at 10.1186/s12962-025-00673-1.

## Introduction

The global economic burden of diabetes is well documented. In 2021, the International Diabetes Federation estimated the global direct costs of diabetes (i.e., the costs of health expenditure due to diabetes) to be $966 billion [1]. Expenditure due to diabetes represents 11.5% of total global health spending, ranging from 8.6% in Europe to 18.4% in South and Central America [1]. In the USA alone, the total cost of diagnosed diabetes (type 1 [T1DM] and type 2 [T2DM] combined) was estimated to be $413 billion in 2022, including $307 billion in direct medical costs and $106 billion in indirect costs [2]. The largest contributor to the direct costs of diabetes is the higher use of prescription medications not directly related to glucose lowering ($84 billion) and of inpatient care ($96 billion) [2]. Reduced employment due to disability ($28 billion), presenteeism ($36 billion) and lost productivity due to premature mortality ($32 billion) are the main drivers of indirect costs [2].

Acute health events directly attributable to diabetes include severe and non-severe hypoglycaemic events, hyperglycaemia, diabetic ketoacidosis (DKA), and coma. Diabetes also increases the risk of developing long-term microvascular (e.g., diabetic nephropathy, neuropathy, and retinopathy) and macrovascular complications (e.g., coronary artery disease, peripheral artery disease, and stroke) [2, 3].

Effective glycaemic control, based on glucose monitoring and self-management by people with diabetes, is important to help reduce the incidence of acute events and the development of complications [4]. Glycaemic control is important even for patients with newly diagnosed T2DM, with glycated haemoglobin (HbA1c) level in the first year after diagnosis having been found to be strongly associated with the risk of future complications [5]. Before the development of sensor-based technologies such as continuous glucose monitoring (CGM) systems, the main form of glucose monitoring was capillary-based self-monitoring of blood glucose (SMBG) using lancets and test strips. SMBG can be burdensome for patients [6, 7], and does not provide comprehensive glucose information [6, 7]. Accordingly, use of CGM technology is becoming common both among people with T1DM and among those with T2DM [8].

The FreeStyle Libre (FSL) systems comprise several iterations of the FSL product, with each iteration adding additional features [9]. The FSL sensors measure glucose in the interstitial fluid every minute [9]. Although the original FSL did not contain alarms to alert users of high or low blood sugar, FSL2 and FSL3 have optional alarms, including a low glucose alarm, high glucose alarm, and signal loss alarm. FSL3 (and, in some countries, FSL2) can also send real-time glucose readings to a smartphone [9].

CGM devices have been shown to improve patients’ glycaemic control in real-world studies and clinical trials [10–18]. One meta-analysis of real-world evidence found FSL to be associated with absolute reductions in HbA1c at 4.5–7.5 months in T1DM (mean, − 0.42%; 95% confidence interval [CI], − 0.58 to − 0.27) and in T2DM (mean, − 0.59%; 95% CI, − 0.80 to − 0.39) [10]. FSL use has been shown to lead to reductions in the proportion of time spent in hypoglycaemia and increases in the time spent in the recommended glycaemic range [11–14]. In addition, FSL has been associated with reductions in inpatient and emergency outpatient treatment due to acute diabetes-related events in large cohort studies in France, Sweden, the UK and the US [16–19], with benefits seen for patients on intensive insulin therapy as well as for those on basal insulin [16, 17].

In addition to potential improvements in patients’ health-related quality of life (HRQoL) and health outcomes, there is potential for cost savings due to the impact of FSL use on acute events (hypoglycaemia and DKA) and long-term complications, which may at least partly offset the acquisition costs of FSL.

The objective of this systematic literature review (SLR) was to identify and synthesize economic evaluations of FSL systems, and to consolidate the available evidence as to the cost effectiveness of FSL systems in different populations. Studies were included that compared FSL with SMBG, other CGM systems or advanced hybrid closed-loop (AHCL) devices, for glucose monitoring by people with either T1DM or T2DM.

## Methods

This systematic review was reported according to the standards of the Preferred Reporting Items for Systematic Review and Meta-Analysis (PRISMA) 2020 Statement [20]. This study was not registered in a public systematic review registry. The full review protocol is available upon request.

### Data sources and search strategy

A PRISMA-compliant search of the published economic evaluations of FSL systems among people with diabetes was conducted in the MEDLINE, Embase, and Cochrane databases. The search strategy (additional file 1: Tables S1 and S2) was developed and tested through an iterative process by a medical information specialist in consultation with the review team. The strategy was peer-reviewed independently by another senior medical information specialist before execution using the Peer Review of Electronic Search Strategies (PRESS) checklist [21]. The search strategy was developed based on the pre-defined Population, Intervention, Comparator, Outcomes, Study Design (PICOS) criteria detailed in additional file 1: Table S3.

Using the Ovid^®^ search interface, the following electronic databases were searched on 27 February 2024: EBM Reviews – Cochrane Central Register of Controlled Trials, EBM Reviews – Cochrane Database of Systematic Reviews, EBM Reviews – Health Technology Assessment, EBM Reviews – NHS Economic Evaluation Database, Embase, Ovid MEDLINE^®^ and Epub Ahead of Print, In-Process, In-Data-Review & Other Non-Indexed Citations and Daily. Vocabulary and syntax were adjusted across databases. English language publications and letters from inception to February 2024 were included. An additional three records were identified by hand searching.

### Screening

Records identified from the electronic database searches were imported into EndNote 20 (Clarivate, Philadelphia, Pennsylvania) and duplicates were removed prior to exporting to the systematic review software (DistillerSR, Evidence Partners, Ottawa, Canada) for study selection. Study selection was conducted by two reviewers who independently reviewed the study records, citation titles, and abstracts to assess eligibility based on the pre-defined PICOS criteria (additional file 1: Table S3). Reviewers documented their reasons for exclusion and any discrepancies between the two reviewers were resolved by consensus or by a third reviewer not involved in the study selection process.

Records considered to describe potentially eligible studies were independently reviewed by two reviewers in full-text form for inclusion in the review. Any discrepancies between the two reviewers were resolved by consensus or by a third reviewer.

### Data extraction and analysis

Data from publications identified in this review were extracted into a standardized form in Microsoft Excel (Microsoft Corporation, Seattle, US). Extracted data were based on the pre-defined PICOS criteria. Data extracted were the study methods, including clinical efficacy, cost and utility inputs, patient characteristics, and the results of the reported cost-effectiveness analyses (CEAs).

To facilitate comparisons between studies, incremental cost-effectiveness ratios (ICERs) and willingness-to-pay (WTP) thresholds were expressed using a common currency. Extracted values were converted into 2024 United States dollars (USD) using the Campbell and Cochrane Economics Methods Group tool as recommended by Cochrane [22, 23], consistent with the approach taken in previous SLRs of economic analyses [24].

For studies describing analyses of FSL Pro, which is intended for use by healthcare professionals, data were extracted but are not described in detail in this review.

### Quality assessment

Quality assessment of the included studies was performed using the Consolidated Health Economic Evaluation Reporting Standards 2022 (CHEERS 2022) checklist [25]. Assessments of quality were performed by one reviewer and validated by a second independent reviewer. Any discrepancies in judgments or quality or justification for judgments between the two reviewers were resolved by consensus or by a third independent reviewer. Overall study quality was represented as the calculated score out of 28 items (or the number of applicable items) expressed as a percentage [26].

## Results

### Search results

**Fig. 1:**
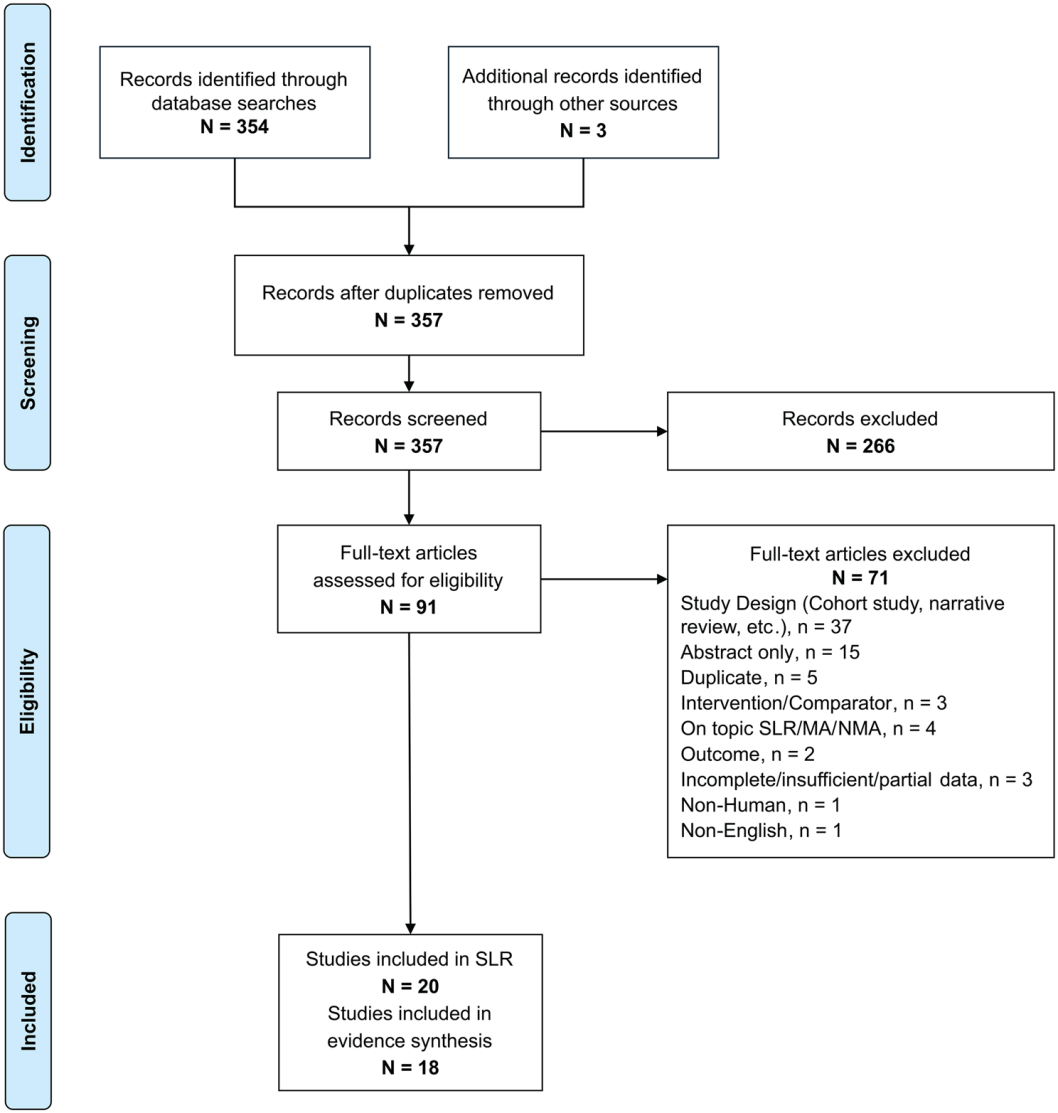
PRISMA diagram. MA, meta-analysis; NMA, network meta-analysis; PRISMA, Preferred Reporting Items for Systematic Reviews and Meta-Analyses; SLR, systematic literature review

The electronic database search identified 354 records, with three additional records identified through other sources. Ninety-one full-text records were retrieved and assessed for eligibility, and 20 were included in the review (Fig. 1) [27–46]. There were no major issues with quality across the included studies (CHEERS mean score, 81%; median score, 80%; additional file 1: Table S4).

### Summary of included studies

This review synthesized evidence from 18 CEAs of FSL [27–44] (Table 1; data from two additional publications describing analyses of FSL Pro are shown in additional file 1: Table S5 [45, 46]).

**Table 1:** Summary of included studies

Study	Country	Treatment	T1DM or T2DM, background therapy	Model (version number)	Outcomes	Funding	Perspective
Ajjan et al. (2022) [27]	UK	FSL vs. SMBG	T2DM, IIT	IQVIA CDM v9.0	LYs, QALYs, total costs, ICER, WTP	Abbott Diabetes Care	Public
Alshannaq et al. (2023) [28]	Denmark	Dexcom G6 vs. FSL and FSL2	T1DM, MDI	IQVIA CDM	Costs, QALYs, ICER, likelihood of cost effectiveness, likelihood of cost savings, WTP	Dexcom	Public
Bahia et al. (2023) [29]	Brazil	FSL vs. SMBG	T1DM and T2DM, IIT	Study’s own decision tree model	QALYs, total costs, ICER, WTP, likelihood of cost effectiveness, NMB	Brazilian Diabetes Society	Public
Bidonde et al. (2017) [30]	Norway	FSL vs. SMBG	T1DM and T2DM, MDI or CSII	IQVIA CDM v9.0	Incremental QALYs, Incremental Costs, ICER	Abbott ^a^	Public
Bilir et al. (2018a) [31]	Sweden	FSL vs. SMBG	T2DM, IIT	IQVIA CDM v8.5	LYs, QALYs, total costs, ICER, WTP	Abbott Diabetes Care	Societal
Bilir et al. (2018b) [32]	Sweden	FSL vs. SMBG	T1DM, IIT	IQVIA CDM v9.0	LYs, QALYs, total costs, ICER, WTP	Abbott Diabetes Care	Public
Elliott et al. (2023) [33]	England	FSL2 vs. SMBG	T1DM, MDI or CSII	IQVIA CDM v9.5	QALYs, total costs, ICER, WTP, likelihood of cost effectiveness	Diabetes UK, NIHR Cambridge Biomedical Research Centre	Public
Emamipour et al. (2024) [34]	Netherlands	FSL vs. before FSL	T1DM, Various therapies	Simulation	Total costs, ICER, WTP, likelihood of cost effectiveness	European Union (Horizon 2020)	Societal
Gardner et al. (2024) [35]	Singapore	MiniMed 780G system (MM780G) vs. FSL plus MDI	T1DM, MDI vs. open-loop systems	IQVIA CDM v9.5	LYs, QALYs, total costs, ICER, WTP, likelihood of cost effectiveness	Medtronic	Public
Isitt et al. (2022) [36]	Australia	Dexcom G6 vs. FSL and SMBG	T1DM, NR	IQVIA CDM	QALYs, total costs, ICER, WTP, likelihood of cost effectiveness	Dexcom	Public
Jendle et al. (2021a) [37]	Sweden	FSL vs. SMBG	T2DM, Insulin	IQVIA CDM v9.5	LYs, QALYs, total costs, ICER, WTP, likelihood of cost effectiveness	Abbott Diabetes Care	Societal
Jendle et al. (2021b) [38]	Sweden	MiniMed 780G system vs. FSL plus MDI	T1DM, MDI or CSII vs. open-loop systems	IQVIA CDM v9.0	LYs, QALYs, total costs, ICER, WTP	Medtronic	Societal
Jendle et al. (2023) [39]	Austria, Greece, Italy, Netherlands, Spain, and Sweden	MiniMed 780G system (MM780G) vs. FSL plus MDI	T1DM, MDI vs. open-loop systems	IQVIA CDM v9.5	QALYs, total costs, ICER	Medtronic	Public
Lambadiari et al. (2022) [40]	Greece	MiniMed 780G system vs. FSL plus MDI	T1DM, MDI vs. open-loop systems	IQVIA CDM	QALYs, total costs, ICER, WTP, likelihood of cost effectiveness	Medtronic	Societal
Rotondi et al. (2022) [41]	Canada	SMBG vs. FSL or Dexcom	T1DM, NR	Study’s own Markov model	QALYs, total costs, ICER, WTP	JDRF Canada	Public
Serne et al. (2022) [42]	Netherlands	MiniMed 670G system vs. FSL plus MDI	T1DM, MDI vs. open-loop systems	IQVIA CDM	Adverse events avoided, QALYs, total costs, ICER, WTP	Medtronic	Societal
Visser et al. (2024) [43]	Belgium	Dexcom G6 vs. FSL	T1DM, MDI or insulin pump	IQVIA CDM v9.0	QALYs, total costs, ICER, WTP, likelihood of cost effectiveness, likelihood of cost savings	Dexcom	Public
Zhao et al. (2021) [44]	China	FSL vs. SMBG	T1DM and T2DM, Basal insulin	IQVIA CDM v9.5	LYs, QALYs, total costs, ICER, WTP, likelihood of cost effectiveness	Abbott Diabetes Care China	Societal for T1DM and Public for T2DM

^a^ The economic analyses considered in the health technology assessment reported by Bidonde et al. (2017) [30] were funded by Abbott

AHCL, advanced hybrid closed-loop; CDM, CORE Diabetes Model; CEA, cost-effectiveness analysis; CSII, continuous subcutaneous insulin infusion; CUA, cost–utility analysis; FSL, FreeStyle Libre; ICER, incremental cost-effectiveness ratio; IIT, intensive insulin therapy; JDRF, Juvenile Diabetes Research Foundation; LY, life year; MDI, multiple daily insulin injections; NA, not applicable; NMB, net monetary benefit; NR, not reported; QALY, quality-adjusted life year; SMBG, self-monitoring blood glucose; T1DM, type 1 diabetes mellitus; T2DM, type 2 diabetes mellitus; UKPDS-OM2, UKPDS Outcomes Model 2; WTP, willingness to pay

## Study perspectives

The majority of the included studies (12/18) took a public healthcare system perspective [27–30, 32, 33, 35, 36, 39, 41, 43, 44]. A total of seven studies [31, 34, 37, 38, 40, 42, 44] reported cost-effectiveness results from a societal perspective (one study reported a societal perspective for T1DM and a public perspective for T2DM [44]).

## Populations investigated

In total, 15 studies reported analyses of FSL in T1DM populations [28–30, 32–36, 38–44], and six described analyses of FSL in T2DM populations [27, 29–31, 37, 44]. Both T1DM and T2DM populations were investigated in three studies [29, 30, 44]. The included studies were mostly conducted from a European perspective [27, 28, 30–34, 37–40, 42, 43], with populations from Australia [36], Brazil [29], Canada [41], China [44], and Singapore [35] also investigated.

## Funding of economic evaluations

In total, six of the included studies were funded by Abbott [27, 30–32, 37, 44]. A further five studies were funded by Medtronic [35, 38–40, 42], and three by Dexcom [28, 36, 43]. The remaining four studies were funded by diabetes organizations [29, 33, 41] and/or research councils [33, 34].

## Devices compared

A total of ten studies reported comparisons between FSL and SMBG [27, 29–34, 37, 41, 44] (T1DM, 7 [29, 30, 32–34, 41, 44], T2DM, 6 [27, 29–31, 37, 44]). Of the remaining studies, all in T1DM populations, three reported comparisons of FSL versus other CGM devices [28, 36, 43], and five compared FSL with AHCL systems [35, 38–40, 42]. All but one of the studies comparing SMBG and FSL assessed the cost effectiveness of FSL; the remaining study considered FSL2 [33]. The three studies comparing with other CGM devices all used FSL [28, 36, 43], with one of these studies also considering FSL2 [28]. All AHCL studies had FSL as the comparator [35, 38–40, 42]. No studies were identified that reported analyses involving FSL3.

## Study outcomes

The main outcome reported was the ICER, described by all 18 studies [27–44], of which 16 also reported local WTP thresholds [27–29, 31–38, 40–44]. Net monetary benefit was reported in one comparison of FSL with SMBG [29].

## Modelling frameworks

Most (15 of 18) of the included studies used the IQVIA CORE Diabetes Model (CDM) [47]. The exceptions were a study comparing FSL with SMBG in Brazil, which used a decision tree model designed for the study [29]; a Canadian study which used a Markov model designed as part of a health technology assessment conducted by Ontario Health [41]; and a Dutch study that estimated the cost effectiveness of FSL using data from a prospective nationwide registry [34].

### Model inputs in the included studies

#### Baseline characteristics

Baseline characteristics and intervention acquisition costs are provided in additional file 1: Table S6. In some studies, baseline characteristics were varied by treatment arm.

#### Glucose monitoring

For FSL, use of 26 sensors per year was considered regardless of diabetes type (with the exception of one study which reported use of 26–29 sensors per year [30]; additional file 1: Table S6). In SMBG-only study arms, SMBG usage was generally higher for people with T1DM (3.5–6 per day) than for people with T2DM (3.0–3.8 per day), with the number of tests per day sourced from local guidelines or clinical trials.

#### Health outcomes

The health impacts of the interventions were measured by improvements in HbA1c, as a surrogate marker of diabetes morbidity, and by reductions in hypoglycaemia and DKA (additional file 1: Table S7). Most of the included studies (16/18) included differences in HbA1c between FSL and the comparator [27, 28, 30, 31, 33–44], with the majority (10/18) incorporating differences in the rate of hypoglycaemia [28, 29, 35, 36, 38, 40–44]. Only five studies included reductions in DKA [29, 35, 40, 41, 44].

#### Utility inputs

Utility differences between FSL and comparators, shown in additional file 1: Table S7, were derived from differences in HbA1c-related complications [27, 28, 30, 33, 35–44] and in the frequency of acute diabetic events (hypoglycaemia and DKA) [27–33, 36, 37, 41, 43, 44]. In addition, treatment-specific utility inputs including avoidance of fingerstick testing and reduction in fear of hypoglycaemia (FoH) were used in most (15/18) studies [27–33, 35–38, 40, 42–44].

#### Results of studies comparing FSL with SMBG

CEA outcomes are shown in detail in additional file 1: Table S8.

#### T1DM populations

**Fig. 2:**
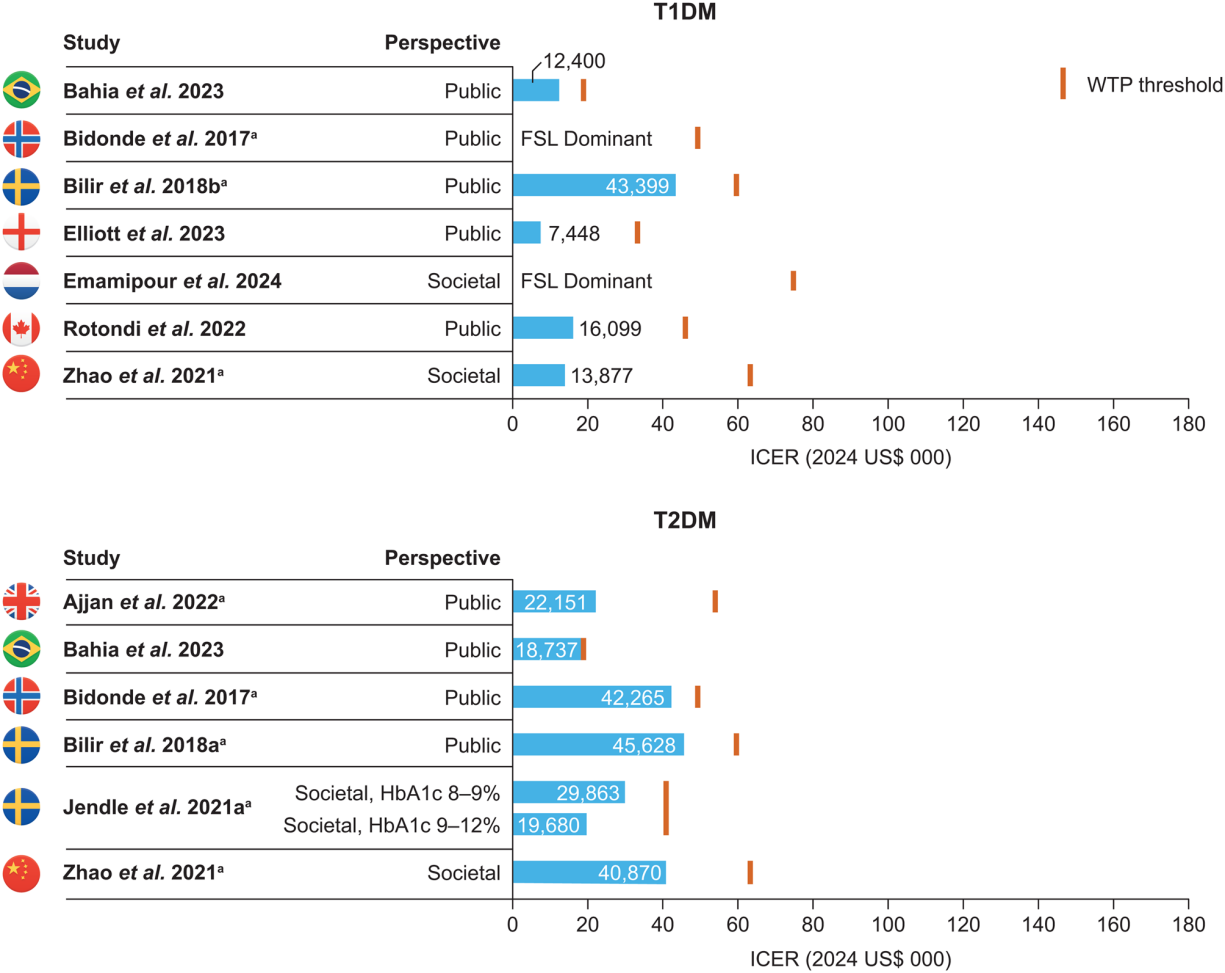
ICERs for FSL vs. SMBG in T1DM or T2DM. All values are shown after conversion to USD 2024. The Norwegian willingness-to-pay threshold shown for Bidonde et al. 2017 [30] is taken from a Norwegian Ministry of Health and Care Services White paper [62] – the reported value of NOK 275,000 in 2017 corresponds to $49,318 in USD 2024. ^a^ Study funded by industry. FSL, FreeStyle Libre; HbA1C, glycated haemoglobin; ICER, incremental cost-effectiveness ratio; SMBG, self-monitoring of blood glucose; T1DM, type 1 diabetes mellitus; T2DM, type 2 diabetes mellitus; WTP, willingness to pay

The seven studies which compared FSL and SMBG in T1DM reported QALY gains ranging from 0.276 to 1.22, resulting in ICERs ranging from FSL being dominant to $43,399 USD/QALY gained (Fig. 2) [29, 30, 32–34, 41, 44]. All seven studies reported that FSL was cost effective under local WTP thresholds [29, 30, 32–34, 41, 44]. In two analyses, FSL was associated with lower costs, compared with SMBG, making it the dominant economic strategy [30, 34]. Elliott et al. (2023) was the only T1DM study not considering FSL, and reported a cost-effective ICER of $7,448 USD/QALY gained for FSL2 versus SMBG in England [33].

#### T2DM populations

The six studies which compared FSL and SMBG in populations of patients with T2DM reported QALY gains ranging from 0.18 to 0.65, and ICERs ranging from $18,737 to $45,628 USD/QALY gained (Fig. 2) [27, 29–31, 37, 44]. Five of these studies reported local WTP thresholds, and FSL was considered cost effective according to these local WTPs in all cases [27, 29, 31, 37, 44]. Zhao et al. (2021) was the only study modelling T2DM treated with basal insulin (as opposed to intensive insulin therapy), and found FSL to be cost effective from a Chinese public payer perspective [44].

#### Net monetary benefit

Bahia et al. (2023) reported that, from the perspective of the Brazilian Public Healthcare System, FSL had a positive net monetary benefit over SMBG (USD, 2024; T1DM, +$1,790; T2DM +$26), indicating that FSL is cost effective [29].

#### Studies not sponsored by industry

Four studies comparing FSL systems and SMBG were not sponsored by industry (Fig. 2) [29, 33, 34, 41]. All of these investigated populations with T1DM [29, 33, 34, 41], and one also assessed a population with T2DM [29]. The four T1DM studies found FSL to be dominant [34] or cost effective at the local WTP threshold, with ICERs of $7,448, $12,400, and $16,099 USD/QALY gained [29, 33, 41]. In the single T2DM study, FSL was cost effective among people using intensive insulin therapy (ICER of $18,737 USD/QALY gained) [29].

### Results of studies comparing FSL with other CGM devices

All three studies that compared FSL with other CGM systems compared the original FSL with the Dexcom G6 device, and considered populations with T1DM (Table 2) [28, 36, 43]. One study also assessed FSL2 (all inputs other than cost were assumed to be the same as for FSL) [28]. The incremental QALY gains associated with CGM were within the range of 0.57 to 1.36, with ICERs ranging from dominant to $13,771 USD/QALY gained [28, 36, 43]. In all three studies, CGM was considered cost effective compared with FSL, with likelihood of cost effectiveness ranging from 76.2% to 89.4% [28, 36, 43]. In Visser et al. (2024), CGM was considered cost-saving compared with FSL and was the dominant economic strategy [43].

**Table 2:** CEA results in studies comparing CGM and AHCL devices with FSL systems

Reference (year)	Intervention vs. comparator	Country, diabetes type, treatment, perspective)	ICER ($/QALY; USD 2024) ^b^	WTP threshold (USD 2024)^b^
*CGM vs. FSL*				
Alshannaq et al. (2023) [28]	Dexcom G6 vs. FSL	Denmark, T1DM, MDI, public	6,146	64,193
	Dexcom G6 vs. FSL2	Denmark, T1DM, MDI, public	5,167	64,193
Isitt et al. (2022) [36]	Dexcom G6 vs. FSL	Australia, T1DM, NR, public	13,771	35,391
Visser et al. (2024) [43]	Dexcom G6 vs. FSL	Belgium, T1DM, MDI or insulin pump, public	Dominant	42,319
AHCL vs. FSL				
Gardner et al. (2024) [35]	MiniMed 780G system/AHCL vs. FSL plus MDI	Singapore, T1DM, MDI vs. open-loop systems, public	25,413	33,837
Jendle et al. (2021b) [38]	MiniMed 780G system/AHCL vs. FSL plus MDI or CSII	Sweden, T1DM, MDI or CSII vs. open-loop systems, societal	49,644	66,422
Jendle et al. (2023) [39]	MiniMed 780G system/AHCL vs. FSL plus MDI	Austria, T1DM, MDI vs. open-loop systems, public	15,801	NA
		Greece, T1DM, MDI vs. open-loop systems, public	24,141	NA
		Italy, T1DM, MDI vs. open-loop systems, public	67,593	NA
		The Netherlands, T1DM, MDI vs. open-loop systems, public	45,402	NA
		Spain, T1DM, MDI vs. open-loop systems, public	50,910	NA
		Sweden, T1DM, MDI vs. open-loop systems, public	56,838	NA
Lambadiari et al. (2022) [40]	MiniMed 780G system/AHCL vs. FSL plus MDI	Greece, T1DM, MDI vs. open-loop systems, societal	60,537	68,909
Serne et al. (2022) [42]	MiniMed 670G system/AHCL vs. FSL plus MDI	Netherlands, T1DM, MDI vs. open-loop systems, societal	9,179	29,934

ICERs are for comparator vs. FSL

CGM, continuous glucose monitoring; CSII, continuous subcutaneous insulin infusion; FSL, FreeStyle Libre; ICER, incremental cost-effectiveness ratio; MDI, multiple daily insulin injections; NR, not reported; QALY, quality-adjusted life year; T1DM, type 1 diabetes mellitus; USD, United States dollar; WTP, willingness to pay

### Results of studies comparing FSL with AHCL

All five studies that compared FSL to AHCL considered populations with T1DM (Table 2) [35, 38–40, 42]. The incremental QALY gains associated with AHCL were within the range of 1.45 to 2.71, with ICERs ranging from $9,179 to $67,593 USD/QALY gained. Four of five studies reported local WTP thresholds, with AHCL considered cost effective compared with FSL in each of these [35, 38, 40, 42].

## Discussion

Comparisons of FSL to SMBG were made in eight countries (Brazil, Canada, China, England, the Netherlands, Norway, Sweden, and UK). FSL was cost effective versus SMBG across all regions, across both T1DM and T2DM, and regardless of the funding source of the study. In some studies, FSL was more cost effective in certain populations, and under certain perspectives.

The results of a Swedish study suggested that for patients with T2DM on insulin, a higher baseline HbA1c was associated with greater FSL cost effectiveness (ICERs by baseline HbA1c: 8–9%, $29,863 USD/QALY gained; 9–12%, $19,680 USD/QALY gained) [37]. This reflects the data used in the model, with a larger reduction in HbA1c applied to patients with higher baseline HbA1c [37]. This correlation between baseline HbA1c and HbA1c reduction following acquisition of FSL has been observed not only in Sweden [48] but also in a meta-analysis of 75 observational studies that found a similar result for both T1DM and T2DM [10]. As with other interventions for diabetes, these results show that patients with poor glycemic control may benefit most from FSL, although it is notable that in the Swedish study FSL was cost effective in both HbA1c subgroups [37].

For studies that reported disease subtype analyses, FSL was more cost effective, compared with SMBG, for patients with T1DM than for those with T2DM [29, 44]. FSL was cost effective for people with T2DM using basal insulin, as well as for those using intensive insulin therapy [44]. In general, the differences in disease-specific cost effectiveness could be explained by demographic and treatment-related factors, not limited to differences in baseline age, HbA1c reductions, severe hypoglycaemia rates, and SMBG testing frequency.

A total of three studies, modelling Australian, Belgian and Danish populations with T1DM, compared FSL with other CGM devices (the comparator was the Dexcom G6 device in each case) [28, 36, 43]. However, although these studies were published after 2022, at which point FSL2 was likely to be the dominant FSL system in use in each country, all three used data for the original FSL system, and not for FSL2 or FSL3 (although one study reported a comparison with FSL2, an assumption was made that all inputs other than cost would be the same as for FSL [28]).

Given that the newer FSL2 and FSL3 devices include improved features such as alarms for low and high glucose and continuous real-time glucose readings sent to smartphones [9], the focus on FSL in these studies may limit their applicability to current decision making. For example, the Dexcom G6 system was modelled as leading to lower mean HbA1c and a lower rate of severe hypoglycaemic events (and FoH) than FSL [28, 36, 43]. However, the difference in performance between the devices was typically attributed to the lack of low and high glucose alarms in FSL, and may not be applicable to FSL2 or FSL3, which have similar alert functions to the Dexcom device. It is notable that the authors of the ALERTT1 study, which was conducted in 2019 and used as a source of clinical inputs in two CEAs included in this review [28, 43], describe the trial as showing the benefits of switching from FSL without alerts to Dexcom G6 with alerts [49]. The authors of the Belgian CEA described in this review also note that the relevance of the comparison between the Dexcom G6 system and the original FSL device may be short-lived due to the development of FSL2 [43].

Recently, two studies of patients with T1DM have found that switching from FSL to FSL2 was also associated with improved time in range, reduced time below range, and a lower average duration of low glucose events [50, 51]. Consistent with this, a recent real-world analysis of diabetes-related events, hospitalizations, and HbA1c reduction found FSL systems and Dexcom G5/G6 systems were associated with similar outcomes for patients with either T1DM or T2DM [52]. It therefore seems likely that in clinical practice recent generations of FSL and other CGM devices have generally similar effectiveness.

FSL and MiniMed AHCL systems were compared in five studies, reporting a total of ten analyses in Singaporean or European populations [35, 38–40, 42]. A limitation of these studies, as for the comparisons with other CGM devices described above, is the use of data for FSL, rather than for FSL2 or FSL3. A further inherent limitation of the published comparisons between FSL and AHCL systems is that the clinical effectiveness of FSL in these analyses relies on patients making insulin therapy decisions based on the information provided by the sensors, rather than the automatic changes in insulin infusion associated with AHCL. The most recent FSL sensor versions can integrate with certain insulin pumps to create a hybrid closed-loop system; further research is needed to evaluate the cost effectiveness of FSL in this configuration.

All but three of the included studies used the IQVIA CDM, which simulates acute diabetes events and long-term complications using a series of Markov models [47, 53]. With multiple studies using similar model structures, differences in results will tend to reflect the model inputs. The effect of modelling assumptions can be particularly important when comparing interventions – in this case sensor-based glucose monitoring technologies – that may have generally similar clinical effectiveness. As described above, the use of data for different generations of device (particularly when new features such as alarms are introduced) is a limitation of several studies included in the review. In addition, the studies comparing FSL with Dexcom G6 and AHCL devices applied utility benefits to FSL and comparator devices in several different ways. In one study, for example, the utility benefit due to avoidance of daily fingerstick testing was taken from a study of FSL, but applied to FSL only at a reduced level [36]. Utility benefits due to reduced FoH were also dealt with inconsistently across studies, with no benefit applied to FSL in several analyses [28, 35, 38, 42, 43]. Given the reductions in FoH seen in real-world studies of FSL [54, 55], this assumption may not be realistic. There is also potential for bias as a result of the choice of clinical data for each comparator. For example, one study used data from a meta-analysis with a moderate–high risk of bias [36, 56], while others compared data from different study types (for example, comparing the results of a small 4-week randomized controlled trial with those of a large 12-month real-world study [38]). Together, these limitations of existing CEAs suggest a need for additional studies to evaluate the cost effectiveness of FSL systems, other CGM devices and AHCL.

Since the systematic searches were conducted, several additional cost-effectiveness studies comparing FSL with SMBG have been published; the results of these studies are consistent with the analyses included in this review [57–61]. A Finnish study based on a real-world cohort of adults with T1DM has found FSL to be cost effective versus SMBG, with an ICER of €9,396/QALY [57]. Similarly, FSL has been found to be dominant to SMBG for T1DM from a Canadian private payer perspective [58]. For people with T2DM, FSL has been found to be cost effective for those using basal insulin in Canada (FSL was dominant) [58], Italy (ICER, €10,556/QALY) [59], or the USA (FSL was dominant) [60]; the Canadian study also found FSL to be dominant to SMBG for people using non-insulin therapies or intensive insulin therapy [58]. A further recent study found that FSL was cost effective from a US payer perspective when added to glucagon-like peptide 1 receptor agonist therapies for the treatment of T2DM, including for patients not using intensive insulin (ICER, $40,968/QALY in the overall cohort; $43,095/QALY in the non-intensive insulin cohort) [61].

This systematic review has some limitations. Most studies comparing FSL systems with SMBG were funded by Abbott, all studies comparing FSL with CGM were funded by Dexcom, and all studies comparing FSL with AHCL were funded by Medtronic. However, all four of the studies comparing FSL and SMBG that were not sponsored by industry reported ICERs within the range of those reported in industry-sponsored studies. Differences in methods, healthcare systems, costs considered, disease characteristics, and background therapies preclude rigorous comparison of the budget impact of FSL across global regions. Similarly, these differences may limit the comparability of outcomes from CEAs.

## Conclusions

Compared with SMBG, FSL systems have demonstrated cost effectiveness in multiple global regions and in populations of patients living with T1DM and T2DM who are using intensive insulin, as well as for those with T2DM who are using basal insulin, across both industry-sponsored and non-sponsored studies. These comparisons were mostly based on the original FSL device, and newer versions may have improved cost effectiveness. Additional studies are needed to validate the cost effectiveness of FSL systems compared with other CGM systems and AHCL devices, considering the range of available evidence, the appropriateness of the compared devices, and the selection of modelling assumptions.

## Supplementary Information

Below is the link to the electronic supplementary material.

Supplementary Material 1

## Data Availability

The dataset supporting the conclusions of this article (i.e., the search strategy and data extracted from the included studies) is included within the article and the additional file.
