# Supplementary material for: Economic analyses of freestyle libre systems for people living with diabetes: a systematic literature review
Source: Cost Eff Resour Alloc. 2025 Nov 19;23:67. doi: 10.1186/s12962-025-00673-1 (PMC12628647; doi:10.1186/s12962-025-00673-1)
Supplement: Supplementary file 1 — Supplementary Material 1 [file 12962_2025_673_MOESM1_ESM.docx]

# Supplementary Tables

**Table S1** Initial search – 17 January 2024 2

**Table S2** Search update – 26 February 2024 6

**Table S3** PICOS criteria 10

**Table S4** Quality assessment of FSL cost-effectiveness analyses 10

**Table S5** Studies of FSL Pro 11

**Table S6** Summary of baseline patient characteristics and acquisition costs 13

**Table S7** Summary of treatment effects and utility benefits considered 17

**Table S8** Summary of cost-effectiveness model outcomes 21

**Table S1** Initial search – 17 January 2024

| **#** | **Searches** | **Results** |
| --- | --- | --- |
| 1 | (((freestyle or free-style or freestyleTM or free-styleTM) adj1 ("libre" or libreTM)) or (("libre" or libretm) adj10 flash) or (flash adj5 glucose) or ((FGM or FGMS or FGMD or FCGM) adj10 glucose) or (("libre" or libreTM) adj2 (pro or proTM or H or HTM or view or viewTM)) or "libreview" or "libreviewTM" or "librepro" or "libreproTM" or "libreH" or "libreHTM" or "Libre 2" or "LibreTM 2" or "LibreTM 2TM" or "Libre 2TM" or Libre2 or Libre2TM or Libre3 or Libre3TM or "Libre 2" or "LibreTM 2" or "LibreTM 2TM" or "Libre 2TM" or isCGM or iscCGM or iCGM or (interstit* adj2 glucose adj2 (monitor* or meter* or sensor*)) or (intermit* adj2 scan* adj3 (glucose or CGM)) or (sensor adj2 based adj2 glucose adj2 (monitor* or meter* or reader* or device*)) or (on adj1 demand adj2 glucose) or (calibrat* adj5 free adj2 glucose) or (factory adj2 calibrat* adj2 glucose) or (("14" or fourteen) adj2 day* adj5 glucose adj2 (monitor* or meter* or device* or reader*)) or ("non" adj1 continuous* adj5 interstitial adj2 glucose) or ((insulinx or insulinxTM) and calculat*) or ((libre or libreTM or librepro or libreproTM) adj5 software*)).ti,ab,sh,hw. | 5148 |
| 2 | ((AGP or "ambulatory glucose" or "glucose profile*") and (libre or flash or FGM or abbott or abbot or abott or FGMD or FGMS)).ti,ab,sh,hw. | 312 |
| 3 | (((glucose adj1 pattern adj1 insight*) or (daily adj1 glucose adj1 summar*) or (daily adj1 pattern*)) and (libre or libreTM)).ti,ab,sh,hw. | 4 |
| 4 | ((libre or libretm or FSL or FSL2 or FSL3) and glucose).ti,ab,sh,hw. | 2457 |
| 5 | ((libre or libretm) and (abbott or abbot or abott)).ti,ab,sh,hw. | 523 |
| 6 | or/1-5 [Free Style Libre device Terms] | 5344 |
| 7 | exp "Costs and Cost Analysis"/ or costs.tw. or cost effective$.tw. [MEDLINE - McM Cost balanced] | 1473763 |
| 8 | (cost$ or cost benefit analys$ or health care costs).mp. [MEDLINE - McM Econ balanced] | 2396462 |
| 9 | Economics/ or exp "Costs and Cost Analysis"/ or Economics, Nursing/ or Economics, Medical/ or exp Economics, Hospital/ or exp "Fees and Charges"/ or exp Budgets/ or exp Models, Economic/ or Markov Chains/ or Monte Carlo Method/ or exp Decision Theory/ or (economic$ or cost or costs or costly or costing or price or prices or pricing or expenditure or expenditures or expense or expenses or financial or finance or finances or financed).ti,kf. or ((cost$ adj2 (effective$ or utilit$ or benefit$ or minimi$ or analy$ or consequence? or outcome or outcomes)) or ((economic or EE) adj3 (model$ or evaluation?))).ab,kf,kw. or ((value adj2 (money or monetary)) or markov or monte carlo or budget$ or (decision$ adj2 (tree$ or analy$ or model$))).ti,ab,kf. | 2474218 |
| 10 | (economic$ or cost or costs or costly or costing or price or prices or pricing or expenditure or expenditures or expense or expenses or financial or finance or finances or financed).ab. /freq=2 | 993930 |
| 11 | 9 or 10 [MEDLINE - CADTH Econ filter - non-validated - MODIFIED] | 2813022 |
| 12 | (Economics/ or exp "Costs and Cost Analysis"/ or exp "Economics, Hospital"/ or Economics, Medical/ or Economics, Nursing/ or (economic$ or cost or costs or costly or costing or price or prices or pricing or (expenditure$ not energy) or (value adj1 money) or budget$).ti,ab.) not (((energy or oxygen) adj cost) or (metabolic adj cost) or ((energy or oxygen) adj expenditure)).ti,ab. [MEDLINE - NHS EED Econ filter - MODIFIED] | 3525646 |
| 13 | 7 or 8 or 11 or 12 [COSTS/ECONOMICS TERMS NOT BURDEN - combined filters - MEDLINE] | 4042805 |
| 14 | 6 and 13 | 563 |
| 15 | exp Animals/ not (exp Animals/ and Humans/) [ANIMAL STUDIES ONLY - REMOVE - MEDLINE] | 16911712 |
| 16 | (address or autobiography or bibliography or biography or dictionary or directory or editorial or "expression of concern" or festschrift or historical article or interactive tutorial or lecture or legal case or legislation or news or newspaper article or patient education handout or personal narrative or portrait or video-audio media or webcast).pt. [Opinion publications - Remove, except for letters/commentaries - MEDLINE] | 2171604 |
| 17 | 14 not (15 or 16) | 536 |
| 18 | 17 use ppez [MEDLINE results] | 128 |
| 19 | (((freestyle or free-style or freestyleTM or free-styleTM) adj1 ("libre" or libreTM)) or (("libre" or libretm) adj10 flash) or (flash adj5 glucose) or ((FGM or FGMS or FGMD or FCGM) adj10 glucose) or (("libre" or libreTM) adj2 (pro or proTM or H or HTM or view or viewTM)) or "libreview" or "libreviewTM" or "librepro" or "libreproTM" or "libreH" or "libreHTM" or "Libre 2" or "LibreTM 2" or "LibreTM 2TM" or "Libre 2TM" or Libre2 or Libre2TM or Libre3 or Libre3TM or "Libre 2" or "LibreTM 2" or "LibreTM 2TM" or "Libre 2TM" or isCGM or iscCGM or iCGM or (interstit* adj2 glucose adj2 (monitor* or meter* or sensor*)) or (intermit* adj2 scan* adj3 (glucose or CGM)) or (sensor adj2 based adj2 glucose adj2 (monitor* or meter* or reader* or device*)) or (on adj1 demand adj2 glucose) or (calibrat* adj5 free adj2 glucose) or (factory adj2 calibrat* adj2 glucose) or (("14" or fourteen) adj2 day* adj5 glucose adj2 (monitor* or meter* or device* or reader*)) or ("non" adj1 continuous* adj5 interstitial adj2 glucose) or ((insulinx or insulinxTM) and calculat*) or ((libre or libreTM or librepro or libreproTM) adj5 software*)).ti,ab,sh,hw,dv. | 5375 |
| 20 | ((AGP or "ambulatory glucose" or "glucose profile*") and (libre or flash or FGM or abbott or abbot or abott or FGMD or FGMS)).ti,ab,sh,hw,dv. | 328 |
| 21 | (((glucose adj1 pattern adj1 insight*) or (daily adj1 glucose adj1 summar*) or (daily adj1 pattern*)) and (libre or libreTM)).ti,ab,sh,hw,dv. | 4 |
| 22 | ((libre or libretm or FSL or FSL2 or FSL3) and glucose).ti,ab,sh,hw,dv. | 2776 |
| 23 | ((libre or libretm) and (abbott or abbot or abott)).ti,ab,sh,hw,dv,tm. | 999 |
| 24 | or/19-23 [Free Style Libre device Terms] | 5580 |
| 25 | (cost or costs).tw. [Embase - Costs McM balanced] | 1818293 |
| 26 | economics/ or cost/ or exp health economics/ or budget/ or statistical model/ or probability/ or monte carlo method/ or decision theory/ or decision tree/ or (economic$ or cost or costs or costly or costing or price or prices or pricing or expenditure or expenditures or expense or expenses or financial or finance or finances or financed).ti,kw. or ((cost$ adj2 (effective$ or utilit$ or benefit$ or minimi$ or analy$ or consequence? or outcome or outcomes)) or ((economic or EE) adj3 (model$ or evaluation?))).ab,kf,kw. or ((value adj2 (money or monetary)) or budget$ or markov or monte carlo or (decision$ adj2 (tree$ or analy$ or model$))).ti,ab,kw. | 4187054 |
| 27 | (economic$ or cost or costs or costly or costing or price or prices or pricing or expenditure or expenditures or expense or expenses or financial or finance or finances or financed).ab. /freq=2 | 993930 |
| 28 | 26 or 27 [Embase CADTH Econ filter - non-validated - MODIFIED] | 4509437 |
| 29 | (health economics/ or exp economic evaluation/ or exp health care cost/ or (econom$ or cost or costs or costly or costing or price or prices or pricing or (expenditure$ not energy) or (value adj2 money) or budget$).ti,ab.) not ((metabolic adj cost) or ((energy or oxygen) adj cost) or ((energy or oxygen) adj expenditure)).ti,ab. [Embase NHS EED Econ filter - MODIFIED] | 3014068 |
| 30 | 25 or 28 or 29 [COSTS/ECONOMICS TERMS NOT BURDEN - combined filters - Embase] | 5646637 |
| 31 | 24 and 30 | 633 |
| 32 | (exp animal/ or exp animal experimentation/ or exp animal model/ or exp animal experiment/ or nonhuman/ or exp vertebrate/) not (exp human/ or exp human experimentation/ or exp human experiment/) [ANIMAL STUDIES ONLY - REMOVE - EMBASE] | 12547301 |
| 33 | (editorial or note or short survey or tombstone).pt. [OPINION PIECES - REMOVE, except for letters - Embase] | 2839532 |
| 34 | 31 not (32 or 33) | 616 |
| 35 | conference abstract.pt. | 5026831 |
| 36 | 34 and 35 [CONFERENCE ABSTRACTS ONLY] | 170 |
| 37 | limit 36 to yr="2022 -Current" | 65 |
| 38 | 34 not 35 [CONFERENCE ABSTRACTS REMOVED] | 446 |
| 39 | 37 or 38 [LAST 2 YRS OF ABSTRACTS RETAINED] | 511 |
| 40 | 39 use oemezd [Embase results] | 295 |
| 41 | (((freestyle or free-style or freestyleTM or free-styleTM) adj1 ("libre" or libreTM)) or (("libre" or libretm) adj10 flash) or (flash adj5 glucose) or ((FGM or FGMS or FGMD or FCGM) adj10 glucose) or (("libre" or libreTM) adj2 (pro or proTM or H or HTM or view or viewTM)) or "libreview" or "libreviewTM" or "librepro" or "libreproTM" or "libreH" or "libreHTM" or "Libre 2" or "LibreTM 2" or "LibreTM 2TM" or "Libre 2TM" or Libre2 or Libre2TM or Libre3 or Libre3TM or "Libre 2" or "LibreTM 2" or "LibreTM 2TM" or "Libre 2TM" or isCGM or iscCGM or iCGM or (interstit* adj2 glucose adj2 (monitor* or meter* or sensor*)) or (intermit* adj2 scan* adj3 (glucose or CGM)) or (sensor adj2 based adj2 glucose adj2 (monitor* or meter* or reader* or device*)) or (on adj1 demand adj2 glucose) or (calibrat* adj5 free adj2 glucose) or (factory adj2 calibrat* adj2 glucose) or (("14" or fourteen) adj2 day* adj5 glucose adj2 (monitor* or meter* or device* or reader*)) or ("non" adj1 continuous* adj5 interstitial adj2 glucose) or ((insulinx or insulinxTM) and calculat*) or ((libre or libreTM or librepro or libreproTM) adj5 software*)).ti,ab,sh,hw. | 5148 |
| 42 | ((AGP or "ambulatory glucose" or "glucose profile*") and (libre or flash or FGM or abbott or abbot or abott or FGMD or FGMS)).ti,ab,sh,hw. | 312 |
| 43 | (((glucose adj1 pattern adj1 insight*) or (daily adj1 glucose adj1 summar*) or (daily adj1 pattern*)) and (libre or libreTM)).ti,ab,sh,hw. | 4 |
| 44 | ((libre or libretm or FSL or FSL2 or FSL3) and glucose).ti,ab,sh,hw. | 2457 |
| 45 | ((libre or libretm) and (abbott or abbot or abott)).ti,ab,sh,hw. | 523 |
| 46 | or/41-45 [Free Style Libre device Terms] | 5344 |
| 47 | exp "costs and cost analysis"/ or costs.tw. or cost effective$.tw. [Translated CENTRAL - McM Cost balanced] | 1473763 |
| 48 | (cost: or cost benefit analys: or health care costs).mp. [Translated CENTRAL - McM Econ balanced] | 2396462 |
| 49 | Economics/ or exp "Costs and Cost Analysis"/ or Economics, Nursing/ or Economics, Medical/ or exp Economics, Hospital/ or exp "Fees and Charges"/ or exp Budgets/ or exp Models, Economic/ or Markov Chains/ or Monte Carlo Method/ or exp Decision Theory/ or (economic$ or cost or costs or costly or costing or price or prices or pricing or expenditure or expenditures or expense or expenses or financial or finance or finances or financed).ti,kw. or ((cost$ adj2 (effective$ or utilit$ or benefit$ or minimi$ or analy$ or consequence? or outcome or outcomes)) or ((economic or EE) adj3 (model$ or evaluation?))).ab,kw. or ((value adj2 (money or monetary)) or markov or monte carlo or budget$ or (decision$ adj2 (tree$ or analy$ or model$))).ti,ab,kw. | 2442850 |
| 50 | (economic$ or cost or costs or costly or costing or price or prices or pricing or expenditure or expenditures or expense or expenses or financial or finance or finances or financed).ab. /freq=2 | 993930 |
| 51 | 49 or 50 [Translated CENTRAL - CADTH Econ filter - non-validated - MODIFIED] | 2792011 |
| 52 | (economics/ or exp "costs and cost analysis"/ or exp "economics, hospital"/ or economics, medical/ or economics, nursing/ or (economic$ or cost or costs or costly or costing or price or prices or pricing or (expenditure$ not energy) or (value adj1 money) or budget$).ti,ab.) not (((energy or oxygen) adj cost) or (metabolic adj cost) or ((energy or oxygen) adj expenditure)).ti,ab. [Translated CENTRAL - NHS EED Econ filter - MODIFIED] | 3525646 |
| 53 | 47 or 48 or 51 or 52 [COSTS/ECONOMICS TERMS NOT BURDEN - combined filters - CENTRAL] | 4030209 |
| 54 | 46 and 53 | 563 |
| 55 | (editorial or note).pt. [OPINION PIECES - REMOVE, except for letters/comments - CENTRAL] | 2455660 |
| 56 | 54 not 55 | 557 |
| 57 | Conference proceeding.pt. [CONFERENCE ABSTRACTS/PROCEEDINGS] | 230423 |
| 58 | 56 and 57 [CONFERENCE ABSTRACTS ONLY] | 13 |
| 59 | limit 58 to yr="2022 -Current" | 7 |
| 60 | 56 not 57 [CONFERENCE ABSTRACTS REMOVED] | 544 |
| 61 | 59 or 60 [LAST 2 YRS OF ABSTRACTS RETAINED] | 551 |
| 62 | 61 use cctr [CENTRAL results] | 58 |
| 63 | (((freestyle or free-style or freestyleTM or free-styleTM) adj1 ("libre" or libreTM)) or (("libre" or libretm) adj10 flash) or (flash adj5 glucose) or ((FGM or FGMS or FGMD or FCGM) adj10 glucose) or (("libre" or libreTM) adj2 (pro or proTM or H or HTM or view or viewTM)) or "libreview" or "libreviewTM" or "librepro" or "libreproTM" or "libreH" or "libreHTM" or "Libre 2" or "LibreTM 2" or "LibreTM 2TM" or "Libre 2TM" or Libre2 or Libre2TM or Libre3 or Libre3TM or "Libre 2" or "LibreTM 2" or "LibreTM 2TM" or "Libre 2TM" or isCGM or iscCGM or iCGM or (interstit* adj2 glucose adj2 (monitor* or meter* or sensor*)) or (intermit* adj2 scan* adj3 (glucose or CGM)) or (sensor adj2 based adj2 glucose adj2 (monitor* or meter* or reader* or device*)) or (on adj1 demand adj2 glucose) or (calibrat* adj5 free adj2 glucose) or (factory adj2 calibrat* adj2 glucose) or (("14" or fourteen) adj2 day* adj5 glucose adj2 (monitor* or meter* or device* or reader*)) or ("non" adj1 continuous* adj5 interstitial adj2 glucose) or ((insulinx or insulinxTM) and calculat*) or ((libre or libreTM or librepro or libreproTM) adj5 software*)).ti,ab,sh,hw. | 5148 |
| 64 | ((AGP or "ambulatory glucose" or "glucose profile*") and (libre or flash or FGM or abbott or abbot or abott or FGMD or FGMS)).ti,ab,sh,hw. | 312 |
| 65 | (((glucose adj1 pattern adj1 insight*) or (daily adj1 glucose adj1 summar*) or (daily adj1 pattern*)) and (libre or libreTM)).ti,ab,sh,hw. | 4 |
| 66 | ((libre or libretm or FSL or FSL2 or FSL3) and glucose).ti,ab,sh,hw. | 2457 |
| 67 | ((libre or libretm) and (abbott or abbot or abott)).ti,ab,sh,hw. | 523 |
| 68 | or/63-67 [Free Style Libre device Terms] | 5344 |
| 69 | 68 use clhta,cleed [HTA, NHS EED results] | 3 |
| 70 | 17 use ppez [MEDLINE results] | 128 |
| 71 | 39 use oemezd [Embase results] | 295 |
| 72 | 61 use cctr [CENTRAL results] | 58 |
| 73 | 63 use coch,clhta,cleed [HTA, NHS EED results] | 3 |
| 74 | 70 or 71 or 72 or 73 [All databases results] | 484 |
| 75 | limit 74 to english language [Limit not valid in CDSR; records were retained] | 474 |
| 76 | limit 75 to yr="1995 -Current" [All databases results - Limited to 1995 - Current & English language - including letters/commentaries from inception] | 345 |
| 77 | remove duplicates from 76 [All databases results - Limited to 1995 - Current & English language - including letters/commentaries from inception - deduplicated] | 345 |

**Table S2** Search update – 26 February 2024

| **#** | **Searches** | **Results** |
| --- | --- | --- |
| 1 | (((freestyle or free-style or freestyleTM or free-styleTM) adj1 ("libre" or libreTM)) or (("libre" or libretm) adj10 flash) or (flash adj5 glucose) or ((FGM or FGMS or FGMD or FCGM) adj10 glucose) or (("libre" or libreTM) adj2 (pro or proTM or H or HTM or view or viewTM)) or "libreview" or "libreviewTM" or "librepro" or "libreproTM" or "libreH" or "libreHTM" or "Libre 2" or "LibreTM 2" or "LibreTM 2TM" or "Libre 2TM" or Libre2 or Libre2TM or Libre3 or Libre3TM or "Libre 2" or "LibreTM 2" or "LibreTM 2TM" or "Libre 2TM" or isCGM or iscCGM or iCGM or (interstit* adj2 glucose adj2 (monitor* or meter* or sensor*)) or (intermit* adj2 scan* adj3 (glucose or CGM)) or (sensor adj2 based adj2 glucose adj2 (monitor* or meter* or reader* or device*)) or (on adj1 demand adj2 glucose) or (calibrat* adj5 free adj2 glucose) or (factory adj2 calibrat* adj2 glucose) or (("14" or fourteen) adj2 day* adj5 glucose adj2 (monitor* or meter* or device* or reader*)) or ("non" adj1 continuous* adj5 interstitial adj2 glucose) or ((insulinx or insulinxTM) and calculat*) or ((libre or libreTM or librepro or libreproTM) adj5 software*)).ti,ab,sh,hw. | 5215 |
| 2 | ((AGP or "ambulatory glucose" or "glucose profile*") and (libre or flash or FGM or abbott or abbot or abott or FGMD or FGMS)).ti,ab,sh,hw. | 321 |
| 3 | (((glucose adj1 pattern adj1 insight*) or (daily adj1 glucose adj1 summar*) or (daily adj1 pattern*)) and (libre or libreTM)).ti,ab,sh,hw. | 4 |
| 4 | ((libre or libretm or FSL or FSL2 or FSL3) and glucose).ti,ab,sh,hw. | 2496 |
| 5 | ((libre or libretm) and (abbott or abbot or abott)).ti,ab,sh,hw. | 529 |
| 6 | or/1-5 [Free Style Libre device Terms] | 5418 |
| 7 | exp "Costs and Cost Analysis"/ or costs.tw. or cost effective$.tw. [MEDLINE - McM Cost balanced] | 1483627 |
| 8 | (cost$ or cost benefit analys$ or health care costs).mp. [MEDLINE - McM Econ balanced] | 2412954 |
| 9 | Economics/ or exp "Costs and Cost Analysis"/ or Economics, Nursing/ or Economics, Medical/ or exp Economics, Hospital/ or exp "Fees and Charges"/ or exp Budgets/ or exp Models, Economic/ or Markov Chains/ or Monte Carlo Method/ or exp Decision Theory/ or (economic$ or cost or costs or costly or costing or price or prices or pricing or expenditure or expenditures or expense or expenses or financial or finance or finances or financed).ti,kf. or ((cost$ adj2 (effective$ or utilit$ or benefit$ or minimi$ or analy$ or consequence? or outcome or outcomes)) or ((economic or EE) adj3 (model$ or evaluation?))).ab,kf,kw. or ((value adj2 (money or monetary)) or markov or monte carlo or budget$ or (decision$ adj2 (tree$ or analy$ or model$))).ti,ab,kf. | 2489102 |
| 10 | (economic$ or cost or costs or costly or costing or price or prices or pricing or expenditure or expenditures or expense or expenses or financial or finance or finances or financed).ab. /freq=2 | 1002022 |
| 11 | 9 or 10 [MEDLINE - CADTH Econ filter - non-validated - MODIFIED] | 2831081 |
| 12 | (Economics/ or exp "Costs and Cost Analysis"/ or exp "Economics, Hospital"/ or Economics, Medical/ or Economics, Nursing/ or (economic$ or cost or costs or costly or costing or price or prices or pricing or (expenditure$ not energy) or (value adj1 money) or budget$).ti,ab.) not (((energy or oxygen) adj cost) or (metabolic adj cost) or ((energy or oxygen) adj expenditure)).ti,ab. [MEDLINE - NHS EED Econ filter - MODIFIED] | 3550354 |
| 13 | 7 or 8 or 11 or 12 [COSTS/ECONOMICS TERMS NOT BURDEN - combined filters - MEDLINE] | 4071315 |
| 14 | 6 and 13 | 574 |
| 15 | exp Animals/ not (exp Animals/ and Humans/) [ANIMAL STUDIES ONLY - REMOVE - MEDLINE] | 16932543 |
| 16 | (address or autobiography or bibliography or biography or dictionary or directory or editorial or "expression of concern" or festschrift or historical article or interactive tutorial or lecture or legal case or legislation or news or newspaper article or patient education handout or personal narrative or portrait or video-audio media or webcast).pt. [Opinion publications - Remove, except for letters/commentaries - MEDLINE] | 2179343 |
| 17 | 14 not (15 or 16) | 546 |
| 18 | 17 use ppez [MEDLINE results] | 132 |
| 19 | (((freestyle or free-style or freestyleTM or free-styleTM) adj1 ("libre" or libreTM)) or (("libre" or libretm) adj10 flash) or (flash adj5 glucose) or ((FGM or FGMS or FGMD or FCGM) adj10 glucose) or (("libre" or libreTM) adj2 (pro or proTM or H or HTM or view or viewTM)) or "libreview" or "libreviewTM" or "librepro" or "libreproTM" or "libreH" or "libreHTM" or "Libre 2" or "LibreTM 2" or "LibreTM 2TM" or "Libre 2TM" or Libre2 or Libre2TM or Libre3 or Libre3TM or "Libre 2" or "LibreTM 2" or "LibreTM 2TM" or "Libre 2TM" or isCGM or iscCGM or iCGM or (interstit* adj2 glucose adj2 (monitor* or meter* or sensor*)) or (intermit* adj2 scan* adj3 (glucose or CGM)) or (sensor adj2 based adj2 glucose adj2 (monitor* or meter* or reader* or device*)) or (on adj1 demand adj2 glucose) or (calibrat* adj5 free adj2 glucose) or (factory adj2 calibrat* adj2 glucose) or (("14" or fourteen) adj2 day* adj5 glucose adj2 (monitor* or meter* or device* or reader*)) or ("non" adj1 continuous* adj5 interstitial adj2 glucose) or ((insulinx or insulinxTM) and calculat*) or ((libre or libreTM or librepro or libreproTM) adj5 software*)).ti,ab,sh,hw,dv. | 5445 |
| 20 | ((AGP or "ambulatory glucose" or "glucose profile*") and (libre or flash or FGM or abbott or abbot or abott or FGMD or FGMS)).ti,ab,sh,hw,dv. | 337 |
| 21 | (((glucose adj1 pattern adj1 insight*) or (daily adj1 glucose adj1 summar*) or (daily adj1 pattern*)) and (libre or libreTM)).ti,ab,sh,hw,dv. | 4 |
| 22 | ((libre or libretm or FSL or FSL2 or FSL3) and glucose).ti,ab,sh,hw,dv. | 2821 |
| 23 | ((libre or libretm) and (abbott or abbot or abott)).ti,ab,sh,hw,dv,tm. | 1022 |
| 24 | or/19-23 [Free Style Libre device Terms] | 5657 |
| 25 | (cost or costs).tw. [Embase - Costs McM balanced] | 1832380 |
| 26 | economics/ or cost/ or exp health economics/ or budget/ or statistical model/ or probability/ or monte carlo method/ or decision theory/ or decision tree/ or (economic$ or cost or costs or costly or costing or price or prices or pricing or expenditure or expenditures or expense or expenses or financial or finance or finances or financed).ti,kw. or ((cost$ adj2 (effective$ or utilit$ or benefit$ or minimi$ or analy$ or consequence? or outcome or outcomes)) or ((economic or EE) adj3 (model$ or evaluation?))).ab,kf,kw. or ((value adj2 (money or monetary)) or budget$ or markov or monte carlo or (decision$ adj2 (tree$ or analy$ or model$))).ti,ab,kw. | 4206114 |
| 27 | (economic$ or cost or costs or costly or costing or price or prices or pricing or expenditure or expenditures or expense or expenses or financial or finance or finances or financed).ab. /freq=2 | 1002022 |
| 28 | 26 or 27 [Embase CADTH Econ filter - non-validated - MODIFIED] | 4531558 |
| 29 | (health economics/ or exp economic evaluation/ or exp health care cost/ or (econom$ or cost or costs or costly or costing or price or prices or pricing or (expenditure$ not energy) or (value adj2 money) or budget$).ti,ab.) not ((metabolic adj cost) or ((energy or oxygen) adj cost) or ((energy or oxygen) adj expenditure)).ti,ab. [Embase NHS EED Econ filter - MODIFIED] | 3037319 |
| 30 | 25 or 28 or 29 [COSTS/ECONOMICS TERMS NOT BURDEN - combined filters - Embase] | 5679283 |
| 31 | 24 and 30 | 644 |
| 32 | (exp animal/ or exp animal experimentation/ or exp animal model/ or exp animal experiment/ or nonhuman/ or exp vertebrate/) not (exp human/ or exp human experimentation/ or exp human experiment/) [ANIMAL STUDIES ONLY - REMOVE - EMBASE] | 12579930 |
| 33 | (editorial or note or short survey or tombstone).pt. [OPINION PIECES - REMOVE, except for letters - Embase] | 2846593 |
| 34 | 31 not (32 or 33) | 626 |
| 35 | conference abstract.pt. | 5061496 |
| 36 | 34 and 35 [CONFERENCE ABSTRACTS ONLY] | 171 |
| 37 | limit 36 to yr="2022 -Current" | 66 |
| 38 | 34 not 35 [CONFERENCE ABSTRACTS REMOVED] | 455 |
| 39 | 37 or 38 [LAST 2 YRS OF ABSTRACTS RETAINED] | 521 |
| 40 | 39 use oemezd [Embase results] | 301 |
| 41 | (((freestyle or free-style or freestyleTM or free-styleTM) adj1 ("libre" or libreTM)) or (("libre" or libretm) adj10 flash) or (flash adj5 glucose) or ((FGM or FGMS or FGMD or FCGM) adj10 glucose) or (("libre" or libreTM) adj2 (pro or proTM or H or HTM or view or viewTM)) or "libreview" or "libreviewTM" or "librepro" or "libreproTM" or "libreH" or "libreHTM" or "Libre 2" or "LibreTM 2" or "LibreTM 2TM" or "Libre 2TM" or Libre2 or Libre2TM or Libre3 or Libre3TM or "Libre 2" or "LibreTM 2" or "LibreTM 2TM" or "Libre 2TM" or isCGM or iscCGM or iCGM or (interstit* adj2 glucose adj2 (monitor* or meter* or sensor*)) or (intermit* adj2 scan* adj3 (glucose or CGM)) or (sensor adj2 based adj2 glucose adj2 (monitor* or meter* or reader* or device*)) or (on adj1 demand adj2 glucose) or (calibrat* adj5 free adj2 glucose) or (factory adj2 calibrat* adj2 glucose) or (("14" or fourteen) adj2 day* adj5 glucose adj2 (monitor* or meter* or device* or reader*)) or ("non" adj1 continuous* adj5 interstitial adj2 glucose) or ((insulinx or insulinxTM) and calculat*) or ((libre or libreTM or librepro or libreproTM) adj5 software*)).ti,ab,sh,hw. | 5215 |
| 42 | ((AGP or "ambulatory glucose" or "glucose profile*") and (libre or flash or FGM or abbott or abbot or abott or FGMD or FGMS)).ti,ab,sh,hw. | 321 |
| 43 | (((glucose adj1 pattern adj1 insight*) or (daily adj1 glucose adj1 summar*) or (daily adj1 pattern*)) and (libre or libreTM)).ti,ab,sh,hw. | 4 |
| 44 | ((libre or libretm or FSL or FSL2 or FSL3) and glucose).ti,ab,sh,hw. | 2496 |
| 45 | ((libre or libretm) and (abbott or abbot or abott)).ti,ab,sh,hw. | 529 |
| 46 | or/41-45 [Free Style Libre device Terms] | 5418 |
| 47 | exp "costs and cost analysis"/ or costs.tw. or cost effective$.tw. [Translated CENTRAL - McM Cost balanced] | 1483627 |
| 48 | (cost: or cost benefit analys: or health care costs).mp. [Translated CENTRAL - McM Econ balanced] | 2412954 |
| 49 | Economics/ or exp "Costs and Cost Analysis"/ or Economics, Nursing/ or Economics, Medical/ or exp Economics, Hospital/ or exp "Fees and Charges"/ or exp Budgets/ or exp Models, Economic/ or Markov Chains/ or Monte Carlo Method/ or exp Decision Theory/ or (economic$ or cost or costs or costly or costing or price or prices or pricing or expenditure or expenditures or expense or expenses or financial or finance or finances or financed).ti,kw. or ((cost$ adj2 (effective$ or utilit$ or benefit$ or minimi$ or analy$ or consequence? or outcome or outcomes)) or ((economic or EE) adj3 (model$ or evaluation?))).ab,kw. or ((value adj2 (money or monetary)) or markov or monte carlo or budget$ or (decision$ adj2 (tree$ or analy$ or model$))).ti,ab,kw. | 2457628 |
| 50 | (economic$ or cost or costs or costly or costing or price or prices or pricing or expenditure or expenditures or expense or expenses or financial or finance or finances or financed).ab. /freq=2 | 1002022 |
| 51 | 49 or 50 [Translated CENTRAL - CADTH Econ filter - non-validated - MODIFIED] | 2810030 |
| 52 | (economics/ or exp "costs and cost analysis"/ or exp "economics, hospital"/ or economics, medical/ or economics, nursing/ or (economic$ or cost or costs or costly or costing or price or prices or pricing or (expenditure$ not energy) or (value adj1 money) or budget$).ti,ab.) not (((energy or oxygen) adj cost) or (metabolic adj cost) or ((energy or oxygen) adj expenditure)).ti,ab. [Translated CENTRAL - NHS EED Econ filter - MODIFIED] | 3550354 |
| 53 | 47 or 48 or 51 or 52 [COSTS/ECONOMICS TERMS NOT BURDEN - combined filters - CENTRAL] | 4058658 |
| 54 | 46 and 53 | 574 |
| 55 | (editorial or note).pt. [OPINION PIECES - REMOVE, except for letters/comments - CENTRAL] | 2461239 |
| 56 | 54 not 55 | 568 |
| 57 | Conference proceeding.pt. [CONFERENCE ABSTRACTS/PROCEEDINGS] | 233853 |
| 58 | 56 and 57 [CONFERENCE ABSTRACTS ONLY] | 13 |
| 59 | limit 58 to yr="2022 -Current" | 7 |
| 60 | 56 not 57 [CONFERENCE ABSTRACTS REMOVED] | 555 |
| 61 | 59 or 60 [LAST 2 YRS OF ABSTRACTS RETAINED] | 562 |
| 62 | 61 use cctr [CENTRAL results] | 59 |
| 63 | (((freestyle or free-style or freestyleTM or free-styleTM) adj1 ("libre" or libreTM)) or (("libre" or libretm) adj10 flash) or (flash adj5 glucose) or ((FGM or FGMS or FGMD or FCGM) adj10 glucose) or (("libre" or libreTM) adj2 (pro or proTM or H or HTM or view or viewTM)) or "libreview" or "libreviewTM" or "librepro" or "libreproTM" or "libreH" or "libreHTM" or "Libre 2" or "LibreTM 2" or "LibreTM 2TM" or "Libre 2TM" or Libre2 or Libre2TM or Libre3 or Libre3TM or "Libre 2" or "LibreTM 2" or "LibreTM 2TM" or "Libre 2TM" or isCGM or iscCGM or iCGM or (interstit* adj2 glucose adj2 (monitor* or meter* or sensor*)) or (intermit* adj2 scan* adj3 (glucose or CGM)) or (sensor adj2 based adj2 glucose adj2 (monitor* or meter* or reader* or device*)) or (on adj1 demand adj2 glucose) or (calibrat* adj5 free adj2 glucose) or (factory adj2 calibrat* adj2 glucose) or (("14" or fourteen) adj2 day* adj5 glucose adj2 (monitor* or meter* or device* or reader*)) or ("non" adj1 continuous* adj5 interstitial adj2 glucose) or ((insulinx or insulinxTM) and calculat*) or ((libre or libreTM or librepro or libreproTM) adj5 software*)).ti,ab,sh,hw. | 5215 |
| 64 | ((AGP or "ambulatory glucose" or "glucose profile*") and (libre or flash or FGM or abbott or abbot or abott or FGMD or FGMS)).ti,ab,sh,hw. | 321 |
| 65 | (((glucose adj1 pattern adj1 insight*) or (daily adj1 glucose adj1 summar*) or (daily adj1 pattern*)) and (libre or libreTM)).ti,ab,sh,hw. | 4 |
| 66 | ((libre or libretm or FSL or FSL2 or FSL3) and glucose).ti,ab,sh,hw. | 2496 |
| 67 | ((libre or libretm) and (abbott or abbot or abott)).ti,ab,sh,hw. | 529 |
| 68 | or/63-67 [Free Style Libre device Terms] | 5418 |
| 69 | 68 use clhta,cleed [HTA, NHS EED results] | 3 |
| 70 | 17 use ppez [MEDLINE results] | 132 |
| 71 | 39 use oemezd [Embase results] | 301 |
| 72 | 61 use cctr [CENTRAL results] | 59 |
| 73 | 63 use coch,clhta,cleed [HTA, NHS EED results] | 3 |
| 74 | 70 or 71 or 72 or 73 [All databases results] | 495 |
| 75 | limit 74 to english language [Limit not valid in CDSR; records were retained] | 485 |
| 76 | limit 75 to yr="1995 -Current" [All databases results - Limited to 1995 - Current & English language - including letters/commentaries from inception] | 485 |
| 77 | remove duplicates from 76 [All databases results - Limited to 1995 - Current & English language - including letters/commentaries from inception - deduplicated] | 351 |

**Table S3** PICOS criteria

| **Criteria** | **Include** | **Exclude** |
| --- | --- | --- |
| Population | Study populations or subgroups of patients (humans only) with any form of diabetes mellitus | Those not listed in the inclusion criteria |
| Intervention | FSL or isCGM or flash glucose monitoring | Those not listed in the inclusion criteria |
| Comparator | Any of:   - Continuous glucose monitor - Self-monitoring of blood glucose - Other glucose monitoring devices | None |
| Outcomes | At least one of the following outcomes:   - Cumulative or incremental costs of intervention - Cumulative or incremental QoL impacts of intervention - Cost effectiveness (expressed as any of cost/adverse event, cost/DALY, cost/QALY, cost/LY, etc.) - Adverse events avoided | Those not listed in the inclusion criteria |
| Study Design | Model-based economic analyses including:   - Cost-effectiveness analysis - Cost–utility analysis - Cost–consequence analysis | Those not listed in the inclusion criteria |
| Location | Global | None |
| Language | Articles in English | All non-English articles |

DALY, disability-adjusted life year; FSL, FreeStyle Libre; isCGM, intermittently scanned continuous glucose monitoring; LY, life year; QALY, quality-adjusted life year; QoL, Quality of Life.

**Table S4** Quality assessment of FSL cost-effectiveness analyses

| **Study** | **Number of applicable CHEERS criteria** | **Number of criteria without issue** | **% Applicable CHEERS criteria met** |
| --- | --- | --- | --- |
| Ajjan *et al*. (2022) [27] | 24 | 22 | 92% |
| Alshannaq *et al*. (2023) [28] | 24 | 18 | 75% |
| Bahia *et al*. (2023) [29] | 25 | 18 | 72% |
| Bidonde *et al*. (2017) [30] | 23 | 18 | 78% |
| Bilir *et al*. (2018a) [31] | 24 | 19 | 79% |
| Bilir *et al*. (2018b) [32] | 24 | 20 | 83% |
| Elliott *et al*. (2023) [33] | 25 | 23 | 92% |
| Emamipour *et al*. (2024) [34] | 24 | 17 | 71% |
| Gardner *et al*. (2024) [35] | 25 | 20 | 80% |
| Isitt *et al*. (2022) [36] | 24 | 19 | 79% |
| Jendle *et al*. (2021a) [37] | 24 | 20 | 83% |
| Jendle *et al*. (2021b) [38] | 24 | 19 | 79% |
| Jendle *et al*. (2023) [39] | 25 | 20 | 80% |
| Lambadiari *et al*. (2022) [40] | 24 | 17 | 71% |
| Rotondi *et al*. (2022) [41] | 24 | 21 | 88% |
| Serne *et al*. (2022) [42] | 24 | 21 | 88% |
| Visser *et al*. (2024) [43] | 24 | 20 | 83% |
| Zhao *et al*. (2021) [44] | 24 | 20 | 83% |

CHEERS, Consolidated Health Economic Evaluation Reporting Standards.

**Table S5** Studies of FSL Pro

| **Study** | **Country** | **Treatment** | | **T1DM or T2DM,**  **background therapy** | | | | **Model (version number)** | | | **Outcomes** | | | | | | **Funding** | | | | **Perspective** | | |
| --- | --- | --- | --- | --- | --- | --- | --- | --- | --- | --- | --- | --- | --- | --- | --- | --- | --- | --- | --- | --- | --- | --- | --- |
| Ajjan *et al*. (2023) [45] | UK | FSL Pro vs. SMBG | | T2DM,  Various therapies | | | | UKPDS-OM2 and Markov Model | | | QALYs, total costs, WTP, likelihood of cost effectiveness, NMB | | | | | | Abbott Diabetes Care | | | | NR | | |
| Hua *et al*. (2021) [46] | Australia | FSL Pro vs. usual clinical care | | T2DM,  Various therapies | | | | UKPDS-OM2 | | | LYs, QALYs, total costs, ICER, WTP | | | | | | National Health and Medical Research Council of Australia  Sanofi Australia  Abbott Diabetes Care | | | | Public | | |
| **Study** | **Treatment arm** | **Diabetes type, therapy** | | **Age, years  (± SD)** | | | | **Sex M/F, %  male** | | | **Duration of diabetes,  years (± SD)** | | | **Baseline HbA1c, %  (± SD)** | | | | **Annual intervention cost** | | | **Cost components** | | |
| Ajjan *et al*. (2023) [45] | FSL Pro | T2DM, various tx | | 62.0 (54.0, 71.0)^a^ | | | | 73.9 | | | 14.5 (0, 20.0)^a^ | | | 9.0 (7.8, 9.7)^a^ | | | | NR | | | NR | | |
|  | SMBG |  |  | 63.0 (53.0, 69.5)^a^ | | | | 72.2 | | | 11.0 (7.0, 17.0)^a^ | | | 8.8 (7.6, 10.7)^a^ | | | | NR | | | NR | | |
| Hua *et al*. (2021) [46] | FSL Pro | T2DM, various tx | | 60.4 (9.8) | | | | 58.40 | | | 13.5 (9.0-20.0)^a^ | | | 8.9 (1.3) | | | | NR | | | 3.5 sensors, 4 GP visits, 1/3 reader, and 1/3 GP training | | |
|  | Usual clinical care |  |  | 59.8 (10.3) | | | | 59.30 | | | 11.0 (8.0-20.0)^a^ | | | 8.9 (1.2) | | | | NR | | | 4 GP visits | | |
| **Study** | **Treatment arm** | **Diabetes type, therapy** | | **Absolute % HbA1c reduction** | | **Source of treatment utility benefit ^a, b, c^** | | | | | | | | | **SHE reduction  (% or RR)** | | | **Rates of SHE** | | **DKA reduction  (% or RR)** | | | **Rates of DKA** |
|  |  |  |  |  |  | HbA1c | | | Event rate | | | Treatment-specific | | |  |  |  |  |  |  |  |  |  |
| Ajjan *et al*. (2023) [45] | FSL Pro | T2DM, various tx | | NR | |  | | | ✓ | | | ✓ | | | NR | | | SHE: 0  SHE2: 1 (over trial period) | | NR | | | NR |
|  | SMBG |  |  | NR | |  | | | ✓ | | |  | | | NR | | | SHE: 2  SHE2: 3 (over trial period) | | NR | | | NR |
| Hua *et al*. (2021) [46] | FSL Pro | T2DM, various tx | | NR | | ✓ | | |  | | |  | | | NR | | | NR | | NR | | | NR |
|  | Usual clinical care |  |  | NR | | ✓ | | |  | | |  | | | NR | | | NR | | NR | | | NR |
| **Study** | **Intervention vs. comparator** | | **Case description (Country, diabetes type, treatment, perspective)** | | **Incremental QALYs** | | **Incremental costs** | | | **ICER (Cost per QALY; Local currency) ^a^** | | | **ICER (cost per QALY; USD 2024) ^b^** | | | **WTP threshold (Local currency)^a^** | | | **WTP threshold (USD 2024)^b^** | | | **Likelihood of cost effectiveness (%)** | |
| Ajjan *et al*. (2023) [45] | FSL Pro vs. SMBG | | UK, T2DM, Various therapies, NR | | NR | | NR | | | Dominant | | | Dominant | | | 20,000 GBP | | | 33,272 | | | 100% | |
| Hua *et al*. (2021) [46] | FSL Pro vs. Usual clinical care | | Australia; T2DM on insulin, public, 3.5 professional FSL tests per year | | 0.03 | | 3807 AUD | | | 120,228 AUD | | | 97,713 | | | 50,000 AUD | | | 40,636 | | | NR | |
|  |  |  | Australia; T2DM on insulin, public, 1.0 professional FSL tests per year | | 0.03 | | 776 AUD | | | 24,493 AUD | | | 19,906 | | | 50,000 AUD | | | 40,636 | | | NR | |

AUD, Australian dollars; FSL, FreeStyle Libre; GBP, British pound sterling; GP, general practitioner; HbA1c, glycated haemoglobin; ICER, incremental cost-effectiveness ratio; LY, life year; NMB, net monetary benefit; NR, not reported; QALY, quality-adjusted life year; RR, relative risk; SD, standard deviation; SHE, severe hypoglycaemic event (general); SHE2, severe hypoglycaemic events 2 (SHEs requiring medical assistance); SMBG, self-monitoring blood glucose; tx, therapy; T2DM, type 2 diabetes mellitus; UKPDS OM2, UKPDS Outcomes Model 2; USD, United States dollar; WTP, willingness to pay

**Table S6** Summary of baseline patient characteristics and acquisition costs

| **Study** | **Treatment arm** | **Diabetes type, therapy** | **Age  (years ± SD)** | **Sex M/F  (% male)** | **Duration of diabetes  (years ± SD)** | **Baseline HbA1c  (%± SD)** | **Annual intervention cost** | **Cost components** |
| --- | --- | --- | --- | --- | --- | --- | --- | --- |
| *FSL vs. SMBG* | | | | | | | | |
| Ajjan *et al*. (2022) [27] | FSL | T2DM, IIT | 66.78 (7.55) | 64.33 | 21.83 | 8.52 | Year 1: 2,432.58 GBP  Year 2+: 2,380.58 GBP | 26 sensors/year, 1 physician visit, 0.3 test strips/day and 0.69 lancets/day, 85.2 units of insulin/day, 1500 mg metformin/day |
|  | SMBG | T2DM, IIT |  |  |  |  | 1,715.81 GBP | 3 test strips/day, 1.26 lancets/day, 87.8 units of insulin/day, 1500 mg metformin/day |
| Bahia *et al*. (2023) [29] | FSL | T1DM, IIT | NR | NR | NR | NR | NR | 1 reader, 1 sensor/14 days, 0.5 SMBG test/day |
|  |  | T2DM, IIT |  |  |  |  | NR | 1 reader, 1 sensor/14 days, 0.3 SMBG test/day |
|  | SMBG | T1DM, IIT | NR | NR | NR | NR | NR | 1 glucometer, 3.5 SMBG tests/day |
|  |  | T2DM, IIT |  |  |  |  | NR | 1 glucometer, 3.5 SMBG tests per day |
| Bidonde *et al*. (2017) [30] | FSL | T1DM, MDI or CSII | 43 (33–57)^a^ | NR | 20 (12-31)^a^ | 6.7 (0.6) | Year 1: 23,446 NOK  Year 2+: 23,162 NOK | 26–29 sensors/year, 2 GP visits, 182.5 SMBG strips/year, 45.8 insulin units/day, 267.4 lancets/year |
|  |  | T2DM, MDI or CSII |  |  |  |  | Year 1: 24,954 NOK  Year 2+: 24,670 NOK | 26–29 sensors/year, 2 GP visits, 109.5 SMBG strips/year, 85.2 insulin units/day, 0.65 lancets/day |
|  | SMBG | T1DM, MDI or CSII | 59 (22–81)^a^ | NR | 17 (2-43)^a^ | 8.74 (1.04) | 14,904 NOK | 1 GP visit, 1,971 SMBG tests/year, 34.8 insulin units/day, 657.6 lancets/year |
|  |  | T2DM, MDI, or CSII |  |  |  |  | 17,116 NOK | 1 GP visit, 3 SMBG strips/day, 87.8 insulin units/day, 1.26 lancets/day |
| Bilir *et al*. (2018a) [31] | FSL | T2DM, IIT | 59.2 (10.3) | 67 | 17.0 (8.0) | 8.68 (1.0) | Year 1: 27,349.70 SEK  Year 2+: 25,923.10 SEK | 109.5 test strips/year, 251.85 lancets/year, 85.2 units of insulin/day, 26 sensors/year, 1 physician visit, 1,500 mg of metformin per day |
|  | SMBG | T2DM, IIT |  |  |  |  | 14,546.97 SEK | 1,095 test strips/year, 459.9 lancets/year, and 87.8 units of insulin/day.1,500 mg of metformin/day |
| Bilir *et al*. (2018b) [32] | FSL | T1DM, IIT | 43.7 (13.9) | 56.9 | 22.0 (12.0) | 6.78 (0.58) | Year 1: 22,142.7 SEK  Year 2+: 20,716.1 SEK | 182.5 test strips/year, 267.4 lancets/year, 45.8 units of insulin/day, 26 sensors/year, 1 physician visit |
|  | SMBG | T1DM, IIT |  |  |  |  | 9,891.46 SEK | 1,971 test strips/year, 657.6 lancets/year, and 38.4 units of insulin/day |
| Elliott *et al*. (2023) [33] | FSL2 | T1DM, MDI, or CSII | 44 (15) | 57 | 20 (11) | NR | Year 1: 1941.16 GBP  Year 2+: 1882.16 GBP | 26 sensors/year, 4 maintenance healthcare visits/year, 0.85 strips/day, 0.85 lancets/day, 0.567 doses insulin/day, 1 GP visit |
|  | SMBG | T1DM, MDI, or CSII | 44(15) | 55 | 20 (11) | NR | 1356.15 GBP | 4.04 strips/day, 4.04 lancets/day, 4 maintenance healthcare visits/year, 52.4 doses insulin/day |
| Emamipour *et al*. (2024) [34] | FSL | T1DM, insulin | 45.6 (15.9) | 50.7 | NR | 7.8 (1.1) | 1669 EUR | NR |
|  | Before FSL | T1DM, insulin |  |  |  |  | NR | NR |
| Jendle *et al*. (2021a) [37] | FSL | T2DM, insulin | 57.1 | 67 | 13.1 | High HbA1c: 8.5 (0.3)  Low HbA1c: 10 (0.9) | Year 1: 22,500 SEK  Year 2+: 21,074 SEK | 0.3 test strips/day, 0.3 lancets/day, 85.2 units of insulin/day, 0.5 reader/year, 26 sensors/year, 1 physician visit,1,500 mg of metformin/day |
|  | SMBG | T2DM, Insulin |  |  |  |  | 12,503 SEK | 3 strips/day, 3 lancets/day, 87.8 units insulin/day, 1,500 mg of metformin/day |
| Rotondi *et al*. (2022) [41] | FSL | T1DM, various tx | NR | NR | NR | NR | 2,540 CAD | 1 reader/ 3 years, 1 sensor/14 days |
|  | SMBG | T1DM, various tx | NR | NR | NR | NR | 2,019 CAD | 1 glucose meter/ 5 years, 6 tests a day |
| Zhao *et al*. (2021) [44] | FSL | T1DM, insulin | 33.3 (13.2) | 49 | 0 (13.2) | 10.3 (0.5) | 13,372 CNY | 0.5 tests/day, insulin, 2.3 sensors |
|  |  | T2DM, basal insulin | 59.4 (10.4) | 55.6 | 9 (6.7) | 8.4 (2.0) | 13,248 CNY | 0.3 tests/day, insulin, 2.3 sensors |
|  | SMBG | T1DM, insulin | 33.3 (13.2) | 49 | 0 (13.2) | 10.3 (0.5) | 3,675 CNY | 5.6 tests/day, insulin |
|  |  | T2DM, basal insulin | 59.4 (10.4) | 55.6 | 9 (6.7) | 8.4 (2.0) | 2,488 CNY | 3.8 tests/day, insulin |
| *FSL vs. CGM* | | | | | | | | |
| Alshannaq *et al*. (2023) [28] | FSL and FSL2 | T1DM, MDI | 42.9 (14.1) | 61.8 | 19.7 (12.9) | 7.4 (0.9) | FSL: 9,713 DKK  FSL2: 10,054 DKK | 1 reader, 26 sensors |
|  | CGM | T1DM, MDI |  |  |  |  | 13,939 DKK | 4 transmitters/year, 36 sensors/year |
| Isitt *et al*. (2022) [36] | FSL | T1DM, NR | 47.6 (12.7) | 56 | 20.3 (13.6) | 8.6 (0.65) | 1,500 AUD | 26 sensors/year, 1 reader/year |
|  | CGM | T1DM, NR |  |  |  |  | 3,200 AUD | 4 transmitters/year, 36 sensors/year |
| Visser *et al*. (2024) [43] | FSL | T1DM, MDI or insulin pump | 42.9 (14.1) | 61.8 | 19.7 (12.9) | 7.4 (0.9) | 1430.80/year | Belgium reimbursement |
|  | CGM | T1DM, MDI or insulin pump |  |  |  |  | 1430.80/year | Belgium reimbursement |
| *FSL vs. AHCL* | | | | | | | | |
| Gardner *et al*. (2024) [35] | FSL | T1DM, MDI vs. open-loop system | 32.2 (15.9) | 24 | NR | 8.4 (1.84) | 3,829 SGD | Insulin, 5 CBG strips/week, 5 lancets/week |
|  | AHCL | T1DM, MDI vs. open-loop system |  |  |  |  | 7,566 SGD | Insulin, 1 pump/4 years, 1 reservoir/3 days, 1 sensor/week |
| Jendle *et al*. (2021b) [38] | FSL | T1DM, MDI or CSII vs. open-loop system | 45.8 (15.3) | 53.9 | 22.8 (13.7) | 7.8 | 29,971.05 SEK | Based on a weighted average of patients in the FUTURE study (77.8% receiving MDI and 22.2% receiving CSII) |
|  | AHCL | T1DM, MDI or CSII vs. open-loop system |  |  |  |  | 75,644.75 SEK | Basal and bolus insulin, CSII pump, cannula, and reservoir, as well as the SMBG testing apparatus and training to use the insulin pump |
| Lambadiari *et al*. (2022) [40] | FSL | T1DM, MDI vs. open-loop system | 45.8 (15.3) | 53.9 | 22.8 (13.7) | 7.8 | 2,940 EUR | 26 sensors/year, short– and long–acting insulin, needles |
|  | AHCL | T1DM, MDI vs. open-loop system |  |  |  |  | 7,507.76 EUR | 1 insulin pump/5 years, 50 sensors/year, short–acting insulin, strips, lancets |
| Serne *et al*. (2022) [42] | FSL | T1DM, MDI vs. open-loop system | 45.8 (15.3) | 53.9 | 22.8 (13.7) | 7.8 (1.2) | 2978.12 EUR | 1 reader, 26 sensors/year, insulin |
|  | AHCL | T1DM, MDI vs. open-loop system |  |  |  |  | 7275.28 EUR | 1 insulin pump/4 years, 52 sensors/year, one Guardian 3 Link kit/year, insulin, strips, lancets |

^a^ Range reported in place of SD

AHCL, advanced hybrid closed-loop; AUD, Australian dollars; CAD, Canadian dollar; CEA, cost-effectiveness analysis; CGM, continuous glucose monitoring; CNY, Chinese Yen; CSII, continuous subcutaneous insulin infusion; CUA, cost–utility analysis; DKK, Danish krone; EUR, Euro; F, female; GBP, British pound sterling; HbA1c, haemoglobin A1C; IIT, intensive insulin therapy; M, male; MDI, multiple daily insulin injections; NOK, Norwegian krone; NR, not reported; RR, relative risk; SD, standard deviation; SEK, Swedish Krone; SGD, Singapore dollar; SMBG, self-monitoring blood glucose; T1DM, type 1 diabetes mellitus; T2DM, type 2 diabetes mellitus; tx, treatment

**Table S7** Summary of treatment effects and utility benefits considered

| **Study** | **Treatment arm** | **Diabetes type, therapy** | **Absolute % HbA1c reduction** | **Source of treatment utility benefit ^a, b, c^** | | | **SHE reduction  (% or RR)** | **Rates of SHE** | **DKA reduction  (% or RR)** | **Rates of DKA** |
| --- | --- | --- | --- | --- | --- | --- | --- | --- | --- | --- |
|  |  |  |  | **HbA1c** | **Event rate** | **Treatment-specific** |  |  |  |  |
| *FSL vs. SMBG* | | | | | | | | | | |
| Ajjan *et al*. (2022) [27] | FSL | T2DM, IIT | −0.85% | ✓ | ✓ | ✓ | SHE2: 0% | SHE2: 105 (per 100 patient‐years) | NR | NR |
|  | SMBG | T2DM, IIT | −0.32% | ✓ | ✓ |  | SHE2: 0% | SHE2: 105 (per 100 patient‐years) | NR | NR |
| Bahia *et al*. (2023) [29] | FSL | T1DM, IIT | NR |  | ✓ | ✓ | NR | 5.05 per patient year | NR | 2.59 (per 100 patient-years) |
|  |  | T2DM, IIT |  |  |  |  | NR | 3.17 per patient year | NR | 0.9 (per 100 patient-years) |
|  | SMBG | T1DM, IIT | NR |  | ✓ |  | NR | 9.8 per patient year | NR | 5.46 (per 100 patient-years) |
|  |  | T2DM, IIT |  |  |  |  | NR | 6.2 per patient year | NR | 1.7 (per 100 patient-years) |
| Bidonde *et al*. (2017) [30] | FSL | T1DM, MDI or CSII | +0.12% | ✓ | ✓ | ✓ | 0% | NR | NR | NR |
|  |  | T2DM, MDI or CSII | −0.29% |  |  |  | 0% | NR | NR | NR |
|  | SMBG | T1DM, MDI or CSII | +0.12% | ✓ | ✓ |  | 0% | NR | NR | NR |
|  |  | T2DM, MDI or CSII | −0.31% |  |  |  | 0% | NR | NR | NR |
| Bilir *et al*. (2018a) [31] | FSL | T2DM, IIT | −0.29% |  | ✓ | ✓ | NR | 105 (per 100 patient‐years) | NR | NR |
|  | SMBG | T2DM, IIT | −0.31% |  | ✓ |  | NR | 105 (per 100 patient‐years) | NR | NR |
| Bilir *et al*. (2018b) [32] | FSL | T1DM, IIT | +0.12% |  | ✓ | ✓ | NR | SHE1: 282.24  SHE2: 37.76  (per 100 patient‐years) | NR | NR |
|  | SMBG | T1DM, IIT | +0.12% |  | ✓ |  | NR | SHE1: 282.24  SHE2: 37.76  (per 100 patient‐years) | NR | NR |
| Elliott *et al*. (2023) [33] | FSL2 | T1DM, MDI or CSII | −0.5% | ✓ | ✓ | ✓ | SHE1: 0%  SHE2: 0% | SHE: 320 (per 100 patient‐years) | NR | NR |
|  | SMBG | T1DM, MDI or CSII | 0% | ✓ | ✓ |  | SHE1: 0%  SHE2: 0% | SHE: 320 (per 100 patient‐years) | NR | NR |
| Emamipour *et al*. (2024) [34] | FSL | T1DM, insulin | −0.35% |  |  |  | NR | NR | NR | NR |
|  | Before FSL | T1DM, insulin | 0% |  |  |  | NR | NR | NR | NR |
| Jendle *et al*. (2021a) [37] | FSL | T2DM, insulin | High HbA1c: −1.3%  Low HbA1c:  −0.41% | ✓ | ✓ | ✓ | NR | SHE1: 0  SHE2: 105  (per 100 patient‐years) | NR | NR |
|  | SMBG | T2DM, insulin | High HbA1c: 0%  Low HbA1c: 0% | ✓ | ✓ |  | NR | SHE1: 0  SHE2: 105  (per 100 patient‐years) | NR | NR |
| Rotondi *et al*. (2022) [41] | FSL | T1DM, various tx | −1.8% | ✓ | ✓ |  | NR | 3.3% per year | NR | 4.3% per year |
|  | SMBG | T1DM, various tx | 0% | ✓ | ✓ |  | NR | 5.2% per year | NR | 6% per year |
| Zhao *et al*. (2021) [44] | FSL | T1DM, insulin | +0.15% | ✓ | ✓ | ✓ | NR | SHE1: 35.7  SHE2: 13.8 (per 100 patient‐years) | NR | 1.0 (per 100 patient‐years) |
|  |  | T2DM, basal insulin | −0.29% |  |  |  | NR | SHE1: 79.4  SHE2: 30.6 (per 100 patient‐years) | NR | NR |
|  | SMBG | T1DM, insulin | +0.17% | ✓ | ✓ |  | NR | SHE1: 43.9  SHE2: 5.9 (per 100 patient‐years) | NR | 1.4 (per 100 patient‐years) |
|  |  | T2DM, basal insulin | −0.31% |  |  |  | NR | SHE1: 92.6  SHE2: 12.4 (per 100 patient‐years) | NR | NR |
| *FSL vs. CGM* | | | | | | | | | | |
| Alshannaq *et al*. (2023) [28] | FSL and FSL2 | T1DM, MDI | 0% | ✓ | ✓ |  | NR | SHE1: 44.3  SHE2: 4.9 (per 100 patient‐years) | NR | NR |
|  | CGM | T1DM, MDI | −0.36% | ✓ | ✓ | ✓ | NR | SHE1: 3.2  SHE2: 1.6 (per 100 patient‐years) | NR | NR |
| Isitt *et al*. (2022) [36] | FSL | T1DM, NR | −0.46% | ✓ | ✓ |  | NR | 4.2 (per 100 patient‐years) | NR | NR |
|  | CGM | T1DM, NR | −1.0% | ✓ | ✓ | ✓ | NR | 3.0 (per 100 patient‐years) | NR | NR |
| Visser *et al*. (2024) [43] | FSL | T1DM, MDI or insulin pump | 0% | ✓ | ✓ |  | NR | SHE1: 44.3  SHE2: 4.9 (per 100 patient‐years) | NR | NR |
|  | CGM | T1DM, MDI or insulin pump | −0.36% | ✓ | ✓ | ✓ | NR | SHE1: 3.2  SHE2: 1.6 (per 100 patient‐years) | NR | NR |
| *FSL vs. AHCL* | | | | | | | | | | |
| Gardner *et al*. (2024) [35] | FSL | T1DM, MDI vs. open-loop system | −0.20% |  |  |  | SHE1: 0%  SHE2: 0% | SHE1: 64.6  SHE2: 13 (per 100 patient‐years) | 0% | 2.93 (per 100 patient‐years) |
|  | AHCL | T1DM, MDI vs. open-loop system | −1.54% |  |  | ✓ | SHE1: 50%  SHE2: 50% | SHE1: 32.3  SHE2: 6.5 (per 100 patient‐years) | 50% | 1.47 (per 100 patient‐years) |
| Jendle *et al*. (2021b) [38] | FSL | T1DM, MDI or CSII vs. open-loop system | 0% |  |  |  | NR | 63.9 (per 100 patient‐years) | NR | 0 (per 100 patient‐years) |
|  | AHCL | T1DM, MDI or CSII vs. open-loop system | −0.5% |  |  | ✓ | NR | 0 (per 100 patient‐years) | NR | 0 (per 100 patient‐years) |
| Jendle *et al*. (2023) [39] | FSL | T1DM, MDI vs. open-loop system | −0.20% | ✓ |  | ✓ | NR | 0 | NR | 0 |
|  | AHCL | T1DM, MDI vs. open-loop system | −1.54% | ✓ |  | ✓ | NR | 0 | NR | 0 |
| Lambadiari *et al*. (2022) [40] | FSL | T1DM, MDI vs. open-loop system | 0% | ✓ |  |  | NR | 63.9 (per 100 patient‐years) | NR | 2.9 (per 100 patient‐years) |
|  | AHCL | T1DM, MDI vs. open-loop system | −0.8% | ✓ |  | ✓ | NR | 0 (per 100 patient‐years) | NR | 0 (per 100 patient‐years) |
| Serne *et al*. (2022) [42] | FSL | T1DM, MDI vs. open-loop system | 0% |  |  |  | NR | 63.9 (per 100 patient‐years) | NR | 0 (per 100 patient‐years) |
|  | AHCL | T1DM, MDI vs. open-loop system | −0.40% |  |  | ✓ | NR | 0 (per 100 patient‐years) | NR | 0 (per 100 patient‐years) |

^a^ HbA1c refers to indirect benefits derived from absolute reductions in HbA1c-related events predicted over the time horizon

^b^ Events relate to utility impact of acute events such as SHE, NSHE and DKA, that are predicted to be unrelated to HbA1c levels

^c^ Treatment-specific considerations include FoH and fingerstick disutility

AHCL, advanced hybrid closed-loop; CEA, cost-effectiveness analysis; CGM, continuous glucose monitoring; CSII, continuous subcutaneous insulin infusion; CUA, cost–utility analysis; DKA, diabetic ketoacidosis; FoH, Fear of hypoglycaemia; HbA1c, haemoglobin A1C; IIT, intensive insulin therapy; MDI, multiple daily insulin injections; NR, not reported; NSHE, non-severe hypoglycaemic event; RR, relative risk; SHE, severe hypoglycaemic event (general); SHE1, severe hypoglycaemic events 1 (SHEs not requiring medical assistance); SHE2, severe hypoglycaemic events 2 (SHEs requiring medical assistance); SMBG, self-monitoring blood glucose; T1DM, type 1 diabetes mellitus; T2DM, type 2 diabetes mellitus; tx, therapy

**Table S8** Summary of cost-effectiveness model outcomes

| **Study** | **Intervention vs. comparator** | **Case description (Country, diabetes type, treatment, perspective)** | **Incremental QALYs** | **Incremental costs** | **ICER (Cost per QALY; Local currency) ^a^** | **ICER (cost per QALY; USD 2024) ^b^** | **WTP threshold (Local currency)^a^** | **WTP threshold (USD 2024)^b^** | **Likelihood of cost effectiveness (%)** |
| --- | --- | --- | --- | --- | --- | --- | --- | --- | --- |
| *FSL vs. SMBG* | | | | | | | | | |
| Ajjan *et al*. (2022) [27] | FSL vs. SMBG | UK, T2DM, IIT, public | NR | NR | 12,309 GBP | 22,151 | 30,000 GBP | 53,988 | NR |
| Bahia *et al*. (2023) [29] | FSL vs. SMBG | Brazil, T1DM, IIT, public | 0.276 | 7,255 BRL | 26,268 BRL | 12,400 | 40,000 BRL | 18,882 | 35.6% |
|  |  | Brazil, T2DM, IIT, public | 0.184 | 7,290 BRL | 39,693 BRL | 18,737 | 40,000 BRL | 18,882 | 42% |
| Bidonde *et al*. (2017) [30] | FSL vs. SMBG | Norway, T1DM, MDI or CSII, public | 1.17 | −1,225,067 NOK | Dominant | Dominant | NR | NA | NR |
|  |  | Norway, T2DM, MDI or CSII, public | 0.38 | 88,731 NOK | 235,673 NOK | 42,265 | NR | NA | NR |
| Bilir *et al*. (2018a) [31] | FSL vs. SMBG | Sweden, T2DM, IIT, societal | 0.56 | 171,192 SEK | 306,082 SEK | 45,628 | 400,000 SEK | 59,628 | NR |
| Bilir *et al*. (2018b) [32] | FSL vs. SMBG | Sweden, T1DM, IIT, public | 0.801 | 233,283 SEK | 291,130 SEK | 43,399 | 400,000 SEK | 59,628 | NR |
| Elliott *et al*. (2023) [33] | FSL2 vs. SMBG | England, T1DM, either on MDI or CSII, public | 0.436 | 1,954 GBP | 4,477 GBP | 7,448 | 20,000 GBP | 33,273 | 95.9% |
| Emamipour *et al*. (2024) [34] | FSL vs. before FSL | Netherlands, T1DM, various tx, societal | NR | NR | Dominant | Dominant | 50,000 EUR | 74,834 | 94% |
| Jendle *et al*. (2021a) [37] | FSL vs. SMBG | Sweden, T2DM, insulin, societal,  Baseline HbA1c: 8–9% | 0.5 | 109,958 SEK | 219,127 SEK | 29,863 | 300,000 SEK | 40,884 | 54% |
|  |  | Sweden, T2DM, insulin, societal,  Baseline HbA1c: 9–12% | 0.57 | 82,171 SEK | 144,412 SEK | 19,680 | 300,000 SEK | 40,884 | 58% |
| Rotondi *et al*. (2022) [41] | FSL vs. SMBG | Canada, T1DM, any background therapy, public | NR | NR | 17,488 CAD | 16,099 | 50,000 CAD | 46,030 | NR |
| Zhao *et al*. (2021) [44] | FSL vs. SMBG | China, T1DM, insulin, societal,  RCT data used | 1.22 | 58,021 CNY | 47,636 CNY | 13,877 | 217,341 CNY | 63,314 | 100% |
|  |  | China, T2DM, basal insulin, public,  RCT data used | 0.65 | 90,997 CNY | 140,297 CNY | 40,870 | 217,341 CNY | 63,314 | 97.2% |
| *FSL vs. CGM ^c^* | | | | | | | | | |
| Alshannaq *et al*. (2023) [28] | Dexcom G6 vs. FSL | Denmark, T1DM, MDI, public,  FSL as comparator | 0.872 | 35,646 DKK | 40,879 DKK | 6,146 | 427,000 DKK | 64,193 | 76.20% |
|  | Dexcom G6 vs. FSL2 | Denmark, T1DM, MDI, public,  FSL2 as comparator | 0.872 | 29,968 DKK | 34,367 DKK | 5,167 | 427,000 DKK | 64,193 | 76.50% |
| Isitt *et al*. (2022) [36] | Dexcom G6 vs. FSL | Australia, T1DM, NR, public | 0.569 | 11,064 AUD | 19,455 AUD | 13,771 | 50,000 AUD | 35,391 | 89.4% |
| Visser *et al*. (2024) [43] | Dexcom G6 vs. FSL | Belgium, T1DM, MDI or insulin pump, public | 1.357 | −8,989 EUR | Dominant | Dominant | 30,000 EUR | 42,319 | 83.5% |
| *FSL vs. AHCL ^c^* | | | | | | | | | |
| Gardner *et al*. (2024) [35] | MiniMed 780G system (MM780G) /AHCL vs. FSL plus MDI | Singapore, T1DM, MDI vs. open-loop systems, public | 1.45 | 36,083 USD | 24,850 USD | 25,413 | 33,087 USD | 33,837 | 81% |
| Jendle *et al*. (2021b) [38] | MiniMed 780G system/AHCL vs. FSL plus MDI or CSII | Sweden, T1DM, MDI or CSII vs. open-loop systems, societal | 1.95 | 727,408 SEK | 373,700 SEK | 49,644 | 500,000 SEK | 66,422 | NR |
| Jendle *et al*. (2023) [39] | MiniMed 780G system (MM780G) /AHCL vs. FSL plus MDI | Austria, T1DM, MDI vs. open-loop systems, public | 2.272 | 26,735 EUR | 11,765 EUR | 15,801 | NR | NA | NR |
|  |  | Greece, T1DM, MDI vs. open-loop systems, public | 2.272 | 29,314 EUR | 12,901 EUR | 24,141 | NR | NA | NR |
|  |  | Italy, T1DM, MDI vs. open-loop systems, public | 2.272 | 99,896 EUR | 43,963 EUR | 67,593 | NR | NA | NR |
|  |  | The Netherlands, T1DM, MDI vs. open-loop systems, public | 2.272 | 74,875 EUR | 32,951 EUR | 45,402 | NR | NA | NR |
|  |  | Spain, T1DM, MDI vs. open-loop systems, public | 2.272 | 67,528 EUR | 29,718 EUR | 50,910 | NR | NA | NR |
|  |  | Sweden, T1DM, MDI vs. open-loop systems, public | 2.272 | 96,358 EUR | 42,405 EUR | 56,838 | NR | NA | NR |
| Lambadiari *et al*. (2022) [40] | MiniMed 780G system/AHCL vs. FSL plus MDI | Greece, T1DM, MDI vs. open-loop systems, societal | 2.708 | 80,880 EUR | 29,869 EUR | 60,537 | 34,000 EUR | 68,909 | 84.2% |
| Serne *et al*. (2022) [42] | MiniMed 670G system/AHCL vs. FSL plus MDI | Netherlands, T1DM, MDI vs. open-loop systems, societal | 2.23 | 13,683 EUR | 6,133 EUR | 9,179 | 20,000 EUR | 29,934 | NR |

^a^ Currency as reported in paper

^b^ Extracted values were converted into 2024 United States dollars (USD) using the Campbell and Cochrane Economics Methods Group tool as recommended by Cochrane [22, 23], consistent with previous SLRs of economic analyses [24]

^c^ ICER is presented as cost effectiveness per QALY over FSL

AUD, Australian dollar; BRL, Brazilian Real; CAD, Canadian dollar; CGM, continuous glucose monitoring; CNY, Chinese yuan renminbi; CSII, continuous subcutaneous insulin infusion; DKK, Danish Krone; EUR, euro; FSL, FreeStyle Libre; GBP, British pound sterling; HbA1c, haemoglobin A1c; ICER, incremental cost-effectiveness ratio; IIT, intensive insulin therapy; MDI, multiple daily insulin injections; NOK, Norwegian Krone; NR, not reported; QALY, quality-adjusted life year; RCT, randomized controlled trial; SEK, Swedish Krona; SMBG, self-monitoring blood glucose; tx, therapy; T1DM, type 1 diabetes mellitus; T2DM, type 2 diabetes mellitus; USD, United States dollar; WTP, willingness to pay
